# A gene trap transposon eliminates haematopoietic expression of zebrafish Gfi1aa, but does not interfere with haematopoiesis

**DOI:** 10.1016/j.ydbio.2016.07.010

**Published:** 2016-09-01

**Authors:** Roshana Thambyrajah, Deniz Ucanok, Maryam Jalali, Yasmin Hough, Robert Neil Wilkinson, Kathryn McMahon, Chris Moore, Martin Gering

**Affiliations:** a School of Life Sciences, University of Nottingham, Queen's Medical Centre, Nottingham NG7 2UH, UK; b Department of Infection, Immunity & Cardiovascular Disease, University of Sheffield, Medical School, Beech Hill Road, Sheffield S10 2RX, UK; c Bateson Centre, University of Sheffield, Firth Court, Western Bank, Sheffield S10 2TN, UK

**Keywords:** Haematopoiesis, Haemogenic endothelium, Endothelial to haematopoietic transition, Zebrafish, Gene trap, Gfi1

## Abstract

A transposon-mediated gene trap screen identified the zebrafish line *qmc551* that expresses a GFP reporter in primitive erythrocytes and also in haemogenic endothelial cells, which give rise to haematopoietic stem and progenitor cells (HSPCs) that seed sites of larval and adult haematopoiesis. The transposon that mediates this GFP expression is located in intron 1 of the *gfi1aa* gene, one of three zebrafish paralogs that encode transcriptional repressors homologous to mammalian Gfi1 and Gfi1b proteins. In *qmc551* transgenics, GFP expression is under the control of the endogenous *gfi1aa* promoter, recapitulates early *gfi1aa* expression and allows live observation of *gfi1aa* promoter activity. While the transposon integration interferes with the expression of *gfi1aa* mRNA in haematopoietic cells, homozygous *qmc551* fish are viable and fertile, and display normal primitive and definitive haematopoiesis. Retained expression of Gfi1b in primitive erythrocytes and up-regulation of Gfi1ab at the onset of definitive haematopoiesis in homozygous *qmc551* carriers, are sufficient to allow normal haematopoiesis. This finding contradicts previously published morpholino data that suggested an essential role for zebrafish Gfi1aa in primitive erythropoiesis.

## Highlights

• A zebrafish gene trap line expresses GFP in primitive and definitive blood cells.
• The gene trap transposon eliminates haematopoietic *gfi1aa* expression.
• Absence of Gfi1aa does not interfere with normal haematopoiesis.
• Gfi1aa and its paralogs Gfi1ab and Gfi1b play redundant roles in haematopoiesis.
• Our data suggest that their roles are conserved in all vertebrates.

## Graphical abstract

**Figure:**
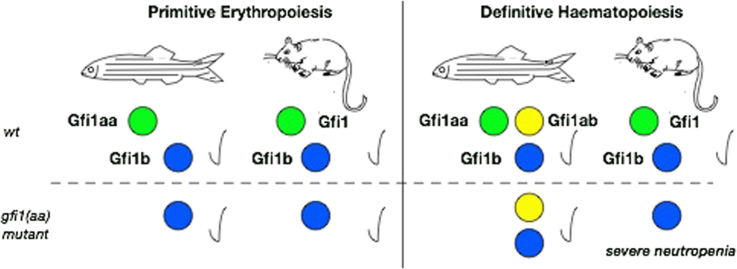

## 1 Introduction

Haematopoietic stem cells (HSCs) are immature blood cells that can self-renew and give rise to mature cells of all blood lineages (Doulatov et al., 2012). HSCs first develop in the embryo. During embryogenesis, haematopoietic cells (HCs) arise in waves from mesodermal progenitors (reviewed in (Ciau-Uitz et al., 2014, Clements and Traver, 2013, Frame et al., 2013, Medvinsky et al., 2011)). In mammals, the first HCs are primitive red blood cells (prRBCs), macrophages and neutrophil granulocytes (Palis et al., 1999, Tober et al., 2007). They develop in the yolk sac from haemangioblasts, mesenchymal cells that are bipotent progenitors for blood and endothelial cells (ECs) (Huber et al., 2004). Haematopoietic progenitor cells (HPCs) and HSCs arise in subsequent waves. They form from haemogenic endothelial cells (HECs) (Chen et al., 2011, Chen et al., 2009, Frame et al., 2016, Yokomizo et al., 2001, Zovein et al., 2008) that undergo endothelial to haematopoietic transition (EHT) and form HC clusters inside the vessels. HPCs develop in yolk sac arteries and veins (Frame et al., 2016) while HSCs form from arterial HECs of the ventral wall of the dorsal aorta (vDA) and from other major arteries (de Bruijn et al., 2000, Gordon-Keylock et al., 2013, Medvinsky and Dzierzak, 1996, Taoudi and Medvinsky, 2007). Explant studies have visualized this process in the mouse vDA (Boisset et al., 2010). Once born, HSCs establish the definitive wave of haematopoiesis that maintains the blood system throughout life.

In zebrafish, primitive blood cells also differentiate from mesenchymal haemangioblasts. The anterior and posterior lateral mesoderm (ALM, PLM) give rise to primitive myeloid and erythroid cells, respectively (Gering et al., 1998, Herbomel et al., 1999, Liao et al., 1998). PLM cells migrate to the midline to form the intermediate cell mass (ICM) where they differentiate into prRBCs, as well as ECs of the DA and the posterior cardinal vein (PCV) (Detrich et al., 1995, Kohli et al., 2013). Primitive erythroblasts first enter circulation between 24 and 25 h post fertilization (hpf). In circulation, they mature over the following days (Qian et al., 2007, Weinstein et al., 1996). After the onset of circulation, definitive HCs begin to arise from HECs in the zebrafish vDA. Unlike in mammals, zebrafish vDA HECs undergo basal epithelial to mesenchymal transition (bEMT) as they turn into HCs (Bertrand et al., 2010, Kissa and Herbomel, 2010, Lam et al., 2010, Zhen et al., 2013). These enter circulation through the vein (Kissa et al., 2008) and seed the caudal haematopoietic tissue (CHT) (Jin et al., 2007, Murayama et al., 2006). The CHT is a transient larval site of haematopoiesis in the tail mesenchyme, where haematopoietic stem and progenitor cells (HSPCs) occupy perivascular niches (Tamplin et al., 2015) before they leave for the kidney and the thymus (Traver et al., 2003).

Gene expression analyses combined with gain- and loss-of-function studies in different vertebrate model organisms have defined sets of transcription factors and co-regulators expressed in developing HCs that tightly regulate gene expression during blood development and, thereby, control cell fate and identity (Orkin and Zon, 2008). The closely related transcriptional repressors Gfi1 and Gfi1b are two of these transcription factors. Gfi1 and Gfi1b play important overlapping roles in adult HSCs and fulfill non-redundant functions in cells of particular blood lineages (Möröy et al., 2015). Gfi1b knockout mice die during gestation with abnormal erythropoiesis and megakaryopoiesis (Saleque et al., 2002). By contrast, Gfi1 knockout mice are viable, but have inner ear defects and severe neutropenia (Hock et al., 2003, Karsunky et al., 2002, Wallis et al., 2003). Gfi1b expressed in place of Gfi1 can substitute for Gfi1 during haematopoiesis, but is not sufficient for normal inner ear development (Fiolka et al., 2006). The zebrafish genome harbors three Gfi1 paralogs (Cooney et al., 2013, Dufourcq et al., 2004, Wei et al., 2008). Two paralogs, *gfi1aa* and *gfi1ab* are orthologs of mammalian *Gfi1,* arisen during a genome duplication in the teleost lineage. The third paralog encodes a Gfi1b protein. Morpholino knockdown studies have indicated that zebrafish *gfi1aa* plays an important role in primitive erythropoiesis (Cooney et al., 2013, Wei et al., 2008) and that *gfi1b* alone is essential for the development of all definitive HC lineages in the embryo (Cooney et al., 2013). While observed defects in definitive erythrocyte and thrombocyte development are consistent with the phenotype of the mouse *gfi1b* knockout, the apparent deficiency in all definitive lineages, as well as the loss of primitive erythropoiesis in the *gfi1aa* morphant embryo were unexpected and suggested a remarkable reshuffling of responsibilities between Gfi1/1b proteins in the bony fish lineage since their divergence from the common teleost and tetrapod ancestor. Here, we report a zebrafish *gfi1aa* gene trap line that demonstrates that the loss of haematopoietic Gfi1aa expression is compatible with primitive erythropoiesis. Our data contradict the previous morpholino studies and suggest that the roles of the mammalian Gfi1 and Gfi1b proteins are conserved in the teleost lineage.

## 2 Results

### 2.1 qmc551:GFP is expressed in primitive erythrocytes and in haemogenic endothelial cells of the dorsal aorta

**Fig. 1:**
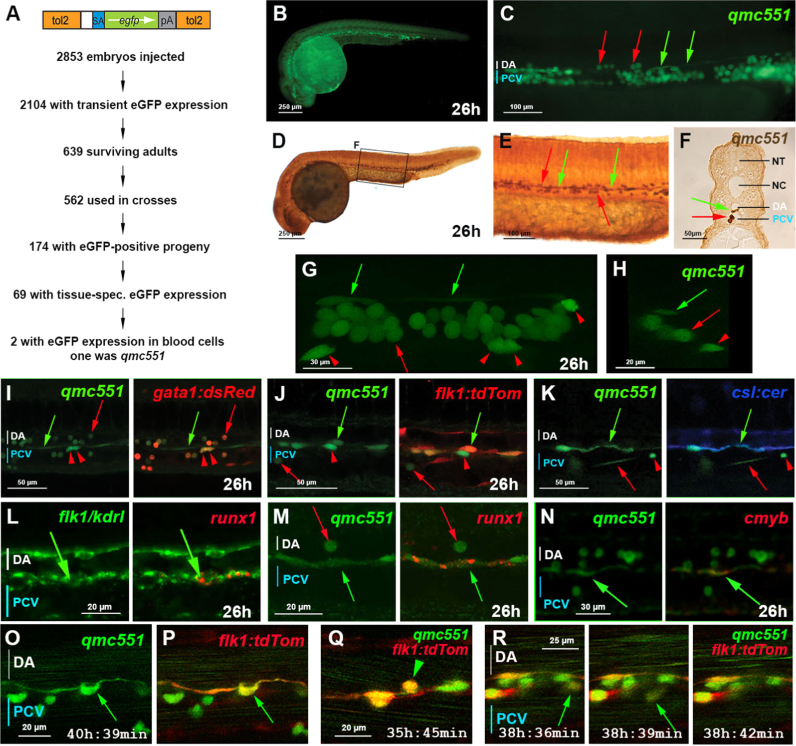
The zebrafish gene trap line *qmc551* expresses GFP in primitive red blood cells and in haemogenic endothelial cells of the ventral wall of the dorsal aorta. (A) Structure of the gene trap transposon and strategy of the gene trap screen. **(B)** Lateral view of a fixed *qmc551* embryo. **(C)** Close-up of the trunk. **(D-F)** GFP immunohistochemistry and diaminobenzidine staining on a *qmc551* embryo. **(E)** A magnified image of the trunk. **(F)** A 10 µm transverse section through the trunk of the embryo after plastic embedding. **(G)** A maximum intensity projection of a 67.5 µm thick confocal Z-stack showing a lateral view of the trunk of a fixed *qmc551* embryo. **(H)** A 1.1 µm YZ cross section of the Z-stack shown in (G). **(I,J)** Confocal images of the trunk of a *qmc551;gata1:dsRed* (I) and *qmc551;flk1:tdTom* (J) double transgenic embryo after fluorescent immunostaining. **(K)** Confocal images of a live *qmc551*;*csl:cer* double transgenic embryo. The *csl:cer* transgene is a derivative of the *csl:venus* transgene which we have previously shown to be expressed in arterial ECs (Gray et al., 2013). **(L)** Double fluorescent *runx1* and *flk1/kdrl* WISH. **(M,N)** Fluorescent *runx1* (M) and *cmyb* (N) WISH combined with GFP immunohistochemistry. **(O-R)** Confocal timelapse microscopy of the DA of *qmc551;flk1:tdTom* embryos. Images were taken every 3 min. Times on panels represent hours and minutes after fertilization. Note that prRBCs in circulation appear as short lines in the confocal image, while stationary cells are round. The confocal analyses in (I-R) were performed at single cell resolution on 2 (I), 1.8 (J), 2.5 (K), 2.0 (L,N), 1.0 (M) and 2.1 (O-R) μm optical slices. All images (B-E,G,I-R) show embryos with anterior to the left and dorsal up. Red arrows – prRBCs; red arrowheads –- prRBC progenitors trapped in the mesenchyme; green arrows – HECs in the vDA.

A transposon-based gene trap approach was used in zebrafish to identify novel genes involved in embryonic haematopoiesis (Fig. 1A). F1 progeny of transposon-injected fish were analyzed for GFP expression at 26 hpf, once most prRBCs had entered circulation. One line, designated *qmc551,* displayed GFP in circulating blood cells and in spindle-shaped cells located between the DA and the PCV (Fig. 1B and C; Movie 1A). Sectioning of GFP-immunostained *qmc551* embryos confirmed GFP expression in blood cells and suggested that the spindle-shaped cells are ECs located in the vDA (Fig. 1D-F). Confocal microscopy revealed additional mesenchymal GFP^+^ cells that appeared to be located just outside the blood vessels (Fig. 1G and H; red arrowheads; Movie 1B). Subsequent examination of double transgenic embryos at single cell resolution in 1.8–2.5 µm optical sections showed that both, the round GFP^+^ cells inside and the mesenchymal cells outside the vessels, co-expressed the prRBC marker gata1: dsRed (Traver et al., 2003), demonstrating that these cells were prRBCs and their precursors, respectively (Fig. 1I/S1A). The spindle-shaped GFP^+^ cells co-expressed the EC reporter flk1: tdTom and were indeed localized in the vDA (Fig. 1J/S1B). Co-expression of the Notch reporter gene *csl: cer* (Fig. 1K/S1C) confirmed these cells were arterial.

Supplementary material related to this article can be found online at 10.1016/j.ydbio.2016.07.010.

The following is the Supplementary material related to this article Movie 1.

Movie 1 : **In the gene trap line *qmc551*, GFP is expressed in primitive red blood cells and in spindle-shaped cells located between the dorsal aorta and the posterior cardinal vein at 26 hpf**. This movie is related to Figure 1. **(A)** Live movie of the trunk and tail of a 26 hpf *qmc551* transgenic. GFP is expressed in circulating and stationary blood cells and in elongated cells located between the dorsal aorta and the posterior cardinal vein. Anterior is to the left, dorsal is up. Pictures of this timelapse movie were taken every 100 ms with a Hamamatsu Orca-ER camera on a Nikon SMZ1500 dissection microscope with an epifluorescence attachment using a FITC filter set. The camera was controlled by IP lab software. Images were pseudo-colored in IP lab and saved as TIFF files. The series of TIFF files was imported into Imaris and turned into a video that was then annotated in iMovie. **(B)** Confocal analysis of the trunk of a fixed 26 hpf *qmc551* transgenic embryo shows expression of GFP in blood cells and in spindle-shaped cells located between the lumen of the dorsal aorta and the lumen of the vein. An animation of a 3D maximum intensity projection of a 67.5 µm thick Z stack is shown. A still image of the maximum intensity projection of the Z stack highlights GFP+endothelial cells (green arrows), red blood cells in circulation (red arrow) and outside the vessels (red arrowheads). The embryo was first treated with an overdose of anesthetic and then fixed in 4% PFA for an hour. The Z-stack was taken on an inverted Zeiss Exciter confocal microscope and has the dimensions 161×161×67.5 µm, respectively. The movie starts with anterior to the left and dorsal up. The confocal images were acquired on a Zeiss Exciter microscope with an EC Plan-Neofluar 40x/1.30 Oil DIC M27 lens..

HECs of the vDA are known to express the stem cell transcription factor Runx1 (Gering and Patient, 2005). Fluorescent whole-mount in situ hybridization (WISH) experiments showed that *runx1* mRNA is localized in foci in *flk1/kdrl*^+^ ECs of the vDA (Fig. 1L/S2A). The same *runx1* mRNA foci were also observed in GFP+ vDA ECs in *qmc551* transgenic embryos (Fig. 1M/S2B). Moreover, a subset of the GFP+ ECs contained *cmyb* mRNA (Fig. 1N/S2C), which is known to be induced downstream of Runx1 (Burns et al., 2005, Kalev-Zylinska et al., 2002). To determine whether these GFP^+^ ECs could undergo EMT to give rise to HCs, confocal timelapse microscopy was performed on *qmc551;flk1/kdrl: tdTom* double transgenic embryos. These experiments revealed that individual ECs with elevated GFP slowly bent towards the mesenchyme, rounded up and eventually joined the mesenchyme as the endothelium closed up above them (Fig. 1O/S2D; Movie 2A). Throughout the process, the cells co-expressed the endothelial reporter transgene (Fig. 1P/S2E; Movie 2B). In addition to bEMT, we also observed that individual GFP^+^ cells left the endothelium apically to enter the DA directly, a process seen only up to 40 hpf (Fig. 1Q/S2F; Movie 3A). Cells, which had undergone bEMT, spent variable periods of time in the mesenchyme before they entered the vein to join the circulation (Fig. 1R/S2G; Movie 3B). These data strongly suggested that qmc551: GFP expression marks HECs prior to, during and after EHT. It is worth noting that the events observed in *qmc551* transgenics were also seen in *flk1/kdrl: gfp;csl:cer* double transgenic embryos (Movies 4 and 5)). Altogether, these data confirmed the erythroid nature of the qmc551: GFP^+^ blood cells and showed that the spindle-shaped GFP^+^ cells were HECs of the vDA. Intrigued by the interesting expression pattern we wanted to know which gene was trapped by the transposon.

Supplementary material related to this article can be found online at 10.1016/j.ydbio.2016.07.010.

The following is the Supplementary material related to this article Movie 2, Movie 3, Movie 4, Movie 5.

Movie 2 : **qmc551: GFP+haemogenic endothelial cells undergo basal epithelial to mesenchymal transition.** This movie is related to Figure 1. It shows image series and still frames of confocal timelapse microscopy experiments performed on *qmc551: eGFP;flk1/kdrl: tdTom*-double transgenic embryos. The embryo was immobilized in 1% low melting point agarose. The embryo faces to the left with dorsal up. The timelapse experiment started at 31 hpf. In (A) only GFP expression is shown, highlighting that qmc551: GFP marks HECs before, during and after epithelial to mesenchymal transition. In (B), both GFP and tdTom fluorescence are shown. tdTom expression confirms that spindle-shaped GFP+ cells undergoing EMT are vDA ECs. tdTom also highlights the outline of the vein. Please note that green horizontal lines in the DA and the PCV are caused by GFP+ prRBCs in circulation. The still frame shows the final image of the timelapse series shown in (B). It highlights the HEC-derived mesenchymal cell (yellow arrow) and the endothelium (white arrow) that has closed up above it. All images show single 2.1 µm optical sections. Images were processed in Imaris and annotated in iMovie..

Movie 3 : **qmc551: GFP+ haemogenic endothelial cells give rise to blood cells that join the circulation.** This movie relates to Figure 1. **(A)** Timelapse movie of 1.5 µm thick optical slices through the trunk of uninjected *qmc551;flk1/kdrl: tdTom* double transgenic embryos from 33 hpf. The movie shows how a double-positive haemogenic endothelial cell leaves the endothelium apically to enter the DA. **(B)** Mesenchymal GFP single (green circle) and GFP/tdTom double-positive (yellow circle) cells migrate through the mesenchyme before they enter the vein to join the circulation. This movie is identical to the one shown in Movie 2, but different cells are highlighted. The cell marked with the yellow circle disappears in Z as it moves from below the vein to a position above the vein. It then re-appears (yellow arrow) before it enters the vein. All images show single 2.1 µm optical sections. Images were processed in Imaris and annotated in iMovie..

Movie 4 : **flk1/kdrl: GFP/csl: Cer-double positive haemogenic endothelial cells give rise to blood cells that join the circulation.** This movie provides supporting evidence related to Figure 1. It shows data from timelapse confocal microscopy on *flk1/kdrl: gfp/csl: cer*-double transgenic embryos starting from 48 hpf. Images of single 1.5 µm optical sections are shown with anterior to the left and dorsal up. Images were taken every 3 min. At 48 hpf, endothelial cells of the dorsal aorta co-express both reporter transgenes, while vein endothelial cells are only GFP-positive. During the course of the timelapse, different events were observed that are all highlighted with colored circles. The red circle marks a haemogenic endothelial cell as it underwent basal endothelial to haematopoietic transition. In the mesenchyme, the cell divided once and the daughter cells remained more or less stationary. As time went on, haematopoietic cells in the mesenchyme lost their green fluorescence faster than their blue fluorescence. Cells that entered the vein to join the circulation were clearly less brightly green than cells that had only just undergone EHT. Based on the residual intensity of their green fluorescence, it was obvious that cells remained in the mesenchyme for different periods of time before they entered the vein. They also entered the vein by different routes. One cell was seen to migrate around the vein before entering it through its ventral wall (blue circle). Another cell entered the vein through its dorsal wall (green circle) while several other cells got trapped in an endothelial pocket formed by venous endothelial cells (yellow circle). The cells eventually left the pocket to join the circulation..

Movie 5 : **Some flk1/kdrl: GFP;csl:Cer double-positive haemogenic endothelial cells leave the endothelium apically.** This movie supports findings reported in Figure 1. It shows data from timelapse confocal microscopy on *flk1/kdrl: gfp/csl: cer*-double transgenic embryos starting from 30 hpf. The arrow points at a haemogenic endothelial cell that leaves the endothelium apically to enter the dorsal aorta. The embryo was immobilized in 1% low melting point agarose. The images show single 1.5 µm thick optical sagittal sections of an embryo with anterior to the left and dorsal up. During the timelapse, images were taken every 3 min..

### 2.2 The gene trapped in *qmc551* is *gfi1aa*

**Fig. 2:**
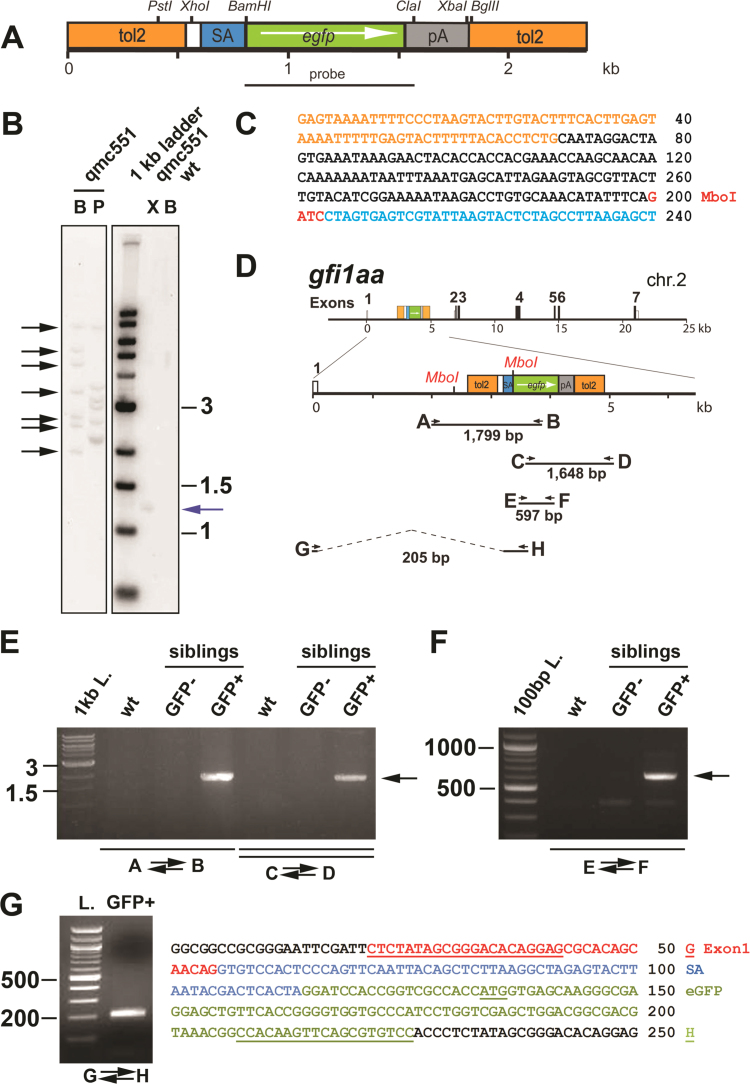
The *qmc551* transposon is located in intron 1 of *gfi1aa* on zebrafish chromosome 2. (A) Gene trap transposon with relevant restriction sites and the probe used in the Southern blot experiment. **(B)** Southern blot experiment on digested genomic DNA isolated from *wt* and *qmc551* transgenic embryos. Black arrows: 7 detected *Bgl*II fragments. Blue arrow: single *XbaI*-*XhoI* band. Restriction enzymes: *BglII* (B), *PstI* (P) or *XbaI* and *XhoI* (X). **(C)** DNA sequence of the nested inverse PCR product. Sequence color code: orange - *tol2*; black - genomic DNA upstream of transposon; blue - splice acceptor. **(D)** Map of the *gfi1aa*^*qmc551Gt*^ locus. The *MboI* sites used in the inverse PCR are highlighted. Positions of oligos used in PCR and RT-PCR experiments are shown and the sizes of the expected PCR products are given. **(E)** PCR amplification across the intron 1/transposon borders on genomic DNA isolated from *wt* embryos, and GFP-negative and GFP+ progeny of a *qmc551* outcross. **(F)** PCR on the same genomic DNAs using *gfp*-internal oligos. **(G)** RT-PCR on total RNA isolated from 3 dpf *qmc551* embryos. Sequence color code: black - vector, red - *gfi1aa* exon 1, blue - splice acceptor; green - GFP. Sequences corresponding to oligos G and H are underlined.

To identify the gene, Southern blot experiments were performed using a probe embedded in the *gfp* gene on the transposon (Fig. 2A). These experiments detected seven copies of the transposon in the genomic DNA of *qmc551* transgenics (Fig. 2B). To reduce the number of genomic integrations in the progeny, outcrosses with wild-type (*wt*) fish were performed. In all of these outcrosses, half of the progeny were GFP^+^ and displayed the full GFP expression pattern, demonstrating that the pattern was not a composite, but reflected GFP expression from a single transposon that was inherited in Mendelian fashion. After 4 generations, nested inverse polymerase chain reaction (PCR) was performed and identified a 134 bp sequence upstream of the transposon (Fig. 2C; for details see Fig. S3) that was identical to a sequence in intron 1 of the zebrafish *gfi1aa* gene (Fig. 2D). This integration site was validated in PCR experiments in which genomic DNA fragments were amplified across both intron 1-transposon boundaries (Fig. 2E). These experiments showed that the GFP expression strictly correlated with the presence of the transposon in *gfi1aa*. Furthermore, PCR experiments with two *gfp*-internal primers, confirmed the absence of silent transposon copies in the *qmc551* line (Fig. 2F). Consistent with the transposon's position, reverse transcription PCR (RT-PCR) allowed successful amplification, cloning and sequencing of a cDNA in which exon 1 of *gfi1aa* was spliced to the splice acceptor on the transposon (Fig. 2G), demonstrating that GFP was transcribed under the control of the *gfi1aa* promoter.

### 2.3 qmc551:GFP reveals endogenous *gfi1aa* expression

**Fig. 3:**
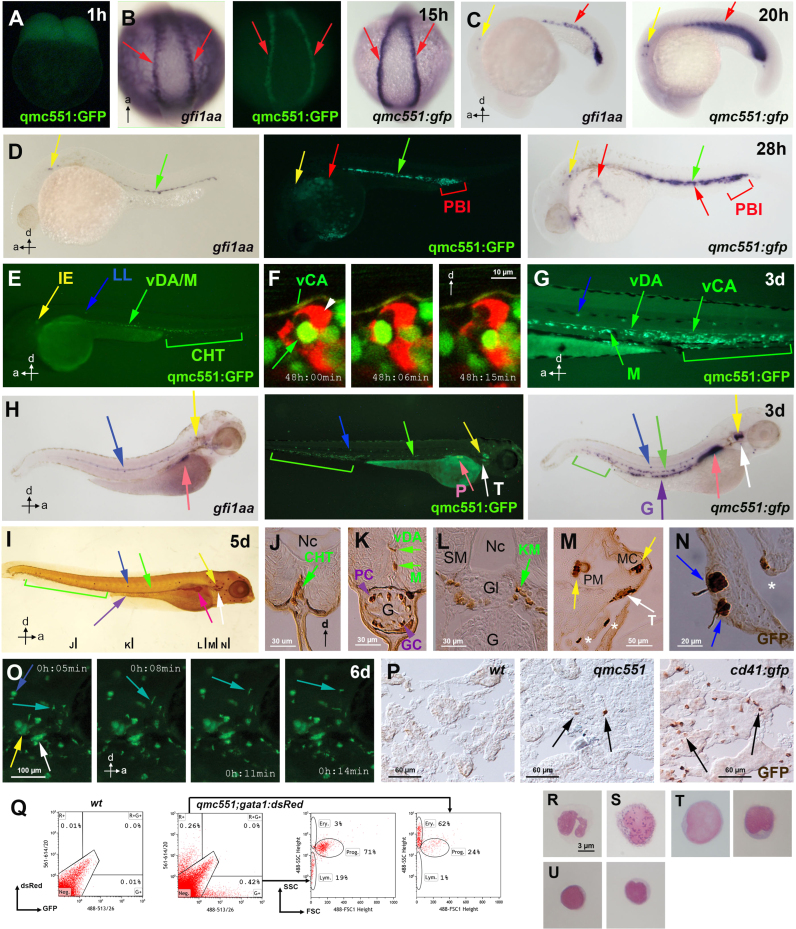
qmc551:GFP expression recapitulates *gfi1aa* expression and marks haematopoietic stem and progenitor cells throughout ontogeny. Views of embryos are posterior in (B) and lateral in (A,C-E,G-I,O). Anteroposterior and dorsoventral axes are indicated. **(A-H)** Images of live *qmc551* embryos and of fixed non-transgenic and *qmc551* transgenic embryos after WISH with probes against endogenous *gfi1aa* and *gfp* mRNA. (F) Confocal timelapse images (2.0 µm thick optical slice) through the CHT of a 48 hpf *qmc551;flk1:tdTom* embryo. **(I)** Images of a *qmc551* transgenic embryo immunostained for GFP expression using diaminobenzidine. **(J-N)**. Transverse 10 µm sections of the same embryo after plastic-embedding. The positions along the anteroposterior axis are indicated in (I). **(O)** Timelapse microscopy of the head region. **(P)** GFP immunohistochemistry and diaminobenzidine staining on 10 µm sections of adult kidneys isolated from *wt*, *qmc551* and *cd41:gfp* fish. **(Q)** Flow cytometric analysis of KM cells of *wt* and *qmc551;gata1:dsRed double* transgenic adults. GFP/dsRed fluorescence and forward/side scatter were analyzed. Excitation and detection wavelengths are indicated in nm. Cell populations were gated according to Traver et al. (2003). **(R-U)** Cytocentrifugation and Giemsa staining of the qmc551:GFP+ KM cells identified neutrophils (R), macrophages (S), progenitors (T) and lymphocytes (U). Annotations: caudal haematopoietic tissue (CHT), glomerulus (Gl), goblet cell (GC), gut (G), inner ear (IE), lateral line organ (LL), medial crista (MC), mesenchyme (M), notochord (Nc), pancreas (P), posterior blood island (PBI), putative Paneth cell (PC), pharyngeal sensory cells (asterisks), posterior macula (PM), swim bladder (SB), somitic muscle (SM), thymus (T) and ventral wall of the DA (vDA) and of the caudal artery (vCA). HSPCs, pRBCs, thymocytes and wandering leukocytes are labeled with green, red, white and turquoise arrows, respectively.

Consistent with its expression under the control of the *gfi1aa* promoter, embryonic GFP mRNA and protein expression patterns in *qmc551* transgenic embryos reflected endogenous *gfi1aa* mRNA expression in non-transgenic embryos. Following early maternal expression (Fig. 3A), zygotic GFP and *gfi1aa* expression was first seen in prRBC progenitors of the PLM (Fig. 3B,C). While *gfi1aa* mRNA diminished in prRBC progenitors between 21 and 28 hpf (Fig. 3C,D), GFP mRNA and protein were still detected in prRBCs in circulation and in the posterior blood island, the posterior extension of the trunk ICM (Fig. 3D). This suggests that GFP mRNA and protein were more stable than endogenous *gfi1aa* mRNA. GFP and *gfi1aa* expression were also found in the inner ear and in ECs of the vDA (Fig. 3D). As GFP^+^ HSPCs left the AGM, they started to accumulate in the CHT where they displayed dynamic interactions with ECs (Fig. 3F, Movie 6A). At 2 and 3 dpf, GFP^+^ ECs expanded posteriorly into the ventral wall of the caudal artery (vCA)(Fig. 3F,G). In addition to the lateral line organ, the exocrine pancreas was seen to express GFP and *gfi1aa* mRNA (Fig. 3H,I). By contrast, only GFP could be detected in cells of the gut, which, based on their position and morphology, as well as in analogy to *Gfi1* expression in the mouse (Bjerknes and Cheng, 2010), are likely to be mucus-filled goblet and crypt-base localized Paneth cells (Fig. 3I and K). While haematopoietic *gfi1aa* expression was undetectable by 3 dpf, GFP mRNA and protein could still be observed in the vDA, in the trunk mesenchyme and in the CHT at 3 and 5 dpf (Fig. 3H–K). In haematopoietic cells, *gfi1aa* mRNA may be present at lower levels that become increasingly difficult to detect by WISH as the larvae grow older. In the live *qmc551* transgenics, GFP^+^ blood cells first seeded the thymus on day 3 (Fig. 3H,I,M) and the larval kidney at day 5 (Fig. 3L). On day 6, wandering GFP^+^ cells were visible throughout the head (Fig. 3O, Movie 6B). These were probably immature leukocytes, since they did not stain with the mature neutrophil marker Sudan black (Le Guyader et al., 2008) and did not co-express the macrophage reporter mpeg1: dsRed (Ellett et al., 2011) (Fig. S4). In adult kidney sections, individual GFP^+^ cells were found between the renal tubules. The sections displayed fewer GFP+ cells than kidney sections of the *cd41: gfp* fish, which are known to express GFP in HSPCs and in cells of the platelet/thrombocyte lineage (Ma et al., 2011) (Fig. 3P). Flow cytometry on kidney marrow (KM) cells of *qmc551;gata1: dsRed* transgenics demonstrated that (a) the GFP^+^ cells fall mainly into the progenitor and lymphoid gates of the forward and side scatter profile (Traver et al., 2003) and (b) do not co-express gata1: dsRed (Fig. 3Q). Thus, while qmc551: GFP was seen in embryonic prRBCs, there was no expression in definitive erythrocytes. Consistent with the flow cytometric data, cytospins of GFP^+^ KM cells identified very few neutrophils with multi-lobed nuclei (Fig. 3R) and macrophages with granules (Fig. 3S). Most of the stained cells were early progenitors with large nuclei and scant cytoplasm (Fig. 3T), and small lymphocytes (Fig. 3U). No erythrocytes were found among the GFP+ cells. Altogether, these data show that qmc551: GFP is not only expressed in HECs that initiate definitive haematopoiesis, but is also found in their HSPC progeny that seed subsequent sites of larval and adult haematopoiesis.

Supplementary material related to this article can be found online at 10.1016/j.ydbio.2016.07.010.

The following is the Supplementary material related to this article Movie 6.

Movie 6 : **qmc551: GFP-positive haematopoietic cells seed perivascular niches in the caudal haematopoietic tissue at 2 dpf and patrol the head tissues at 6 dpf.** This movie is related to Figure 3. **(A)** Timelapse confocal microscopy on *qmc551;flk1: tdTom* double transgenic embryos starting from 48 hpf showing 2.0 µm thick optical slices through the caudal haematopoietic tissue. Here, GFP+ haematopoietic cells undergo dynamic interactions with tdTom+ ECs. One of the cells is highlighted with a yellow circle. Please note that ECs in the ventral wall of the caudal artery co-express the two transgenes. Anterior is to the left, dorsal is up. The embryo shown was immobilized in 1% low melting point agarose. **(B)** A 6 dpf *qmc551* transgenic embryo was anesthetized and placed in a tiny depression in agarose under a fluorescent Nikon SMZ1500 dissection microscope using a Nikon DS-5Mc/DS-U1 camera setup. From minute 5 on, pictures of the head region of the embryo (facing right) were taken manually every 3 min. Images were imported into Photoshop. In Photoshop, the pictures were moved and rotated to correct for the drifting movement of the embryo under the microscope. Furthermore, annotations were added. All pictures were then imported into iMovie to generate the final video. Individual frames are also shown in Figure 3O..

### 2.4 Haemogenic endothelial qmc551:GFP expression is induced in parallel to Runx1 downstream of Vegf and Notch signaling

**Fig. 4:**
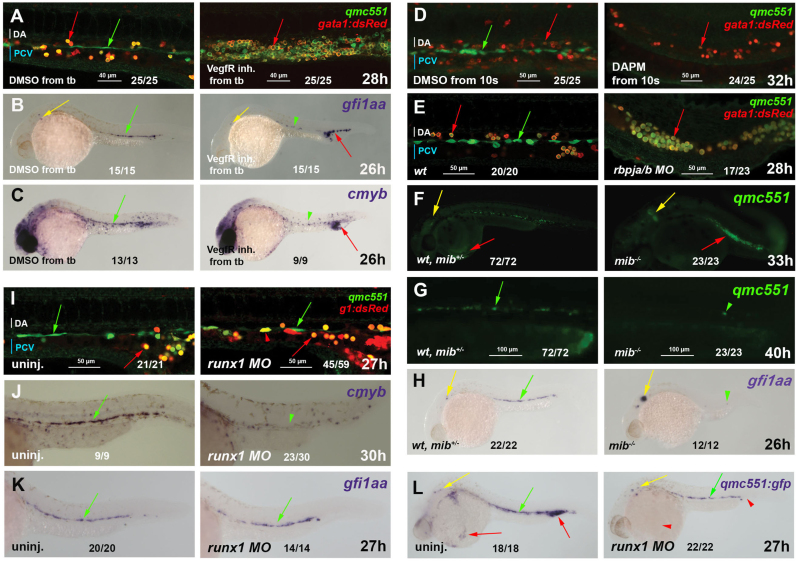
*Gfi1aa* expression in haemogenic endothelial cells is induced downstream of VegfA and Notch signaling, but independent of Runx1. Fixed *qmc551;gata1:dsRed* double transgenic embryos after GFP/dsRed immunohistochemistry are shown in **(A, D-E, I)**. Fixed *wt*, *qmc551* and *qmc551;gata1:dsRed* double transgenic embryos stained by WISH are shown in **(B-C, H, K)**, **(L)** and **(J)**, respectively. Live *qmc551* embryos that were *wt*, heterozygous or homozygous *mib* carriers were imaged in **(F-G)**. Confocal images of optical sagittal sections through the DA are 1.6 and 0.995 µm (A), 6.5 and 6.6 µm (D), 1.2 µm (E) and 2.7 µm (I) thick. A confocal maximum intensity projection of a 37 µm optical slice is shown in (G). Embryos were treated with DMSO, the VegfR inhibitors 676,475 (A) and SU5416 (B,C) or DAPM (D) from tailbud stage (10 hpf). *Rbpja/b* (E) and *runx1* (I-L) morpholinos were injected at 2–4 cell stage. PrRBCs, HECs and inner ear hair cells are labeled with red, green and yellow arrows, respectively. Arrowheads mark reduced or absent staining. Fractions x/y give the number of embryos, x, with depicted phenotype out of all embryos analyzed, y. Embryos are shown with anterior left and dorsal up.

It had previously been shown that *gfi1aa* expression in prRBCs occurs downstream of *cloche* and *scl/tal1*, but is independent of *gata1* and *frs* (Cooney et al., 2013). Here, we focused on the regulation of *gfi1aa* in HECs of the vDA. In HECs, *runx1* expression is known to be induced downstream of a signaling cascade that includes Hedgehog, VegfA and Notch signaling (Burns et al., 2005, Gering and Patient, 2005, Rowlinson and Gering, 2010). We, therefore, tested whether qmc551: GFP and *gfi1aa* expression in HECs also required Vegf and Notch signaling. Treatment of double transgenic embryos with a VegfR inhibitor led to the pooling of qmc551: GFP+/gata1: dsRed+ prRBCs in the ICM and to a complete loss of GFP^+^ spindle-shaped ECs (Fig. 4A). Likewise, non-transgenic embryos lacked all *gfi1aa* and *cmyb* mRNA in the trunk HECs in the absence of Vegf signaling (Fig. 4B and C).

To determine whether *gfi1aa* expression in the vDA required the Notch pathway, *qmc551;gata1: dsRed* embryos were either treated with the γ-secretase inhibitor DAPM (Geling et al., 2002) or injected with the Rbpja/b morpholino (Sieger et al., 2003). The *qmc551* transgene was also crossed into the *mindbomb* (*mib*^*ta52b*^) (Itoh et al., 2003) mutant background. All three types of Notch-depleted embryos specified GFP^+^ prRBCs, but lacked GFP^+^ HECs (Fig. 4D-G). A WISH experiment performed on non-transgenic *mib* mutants confirmed the loss of *gfi1aa* expression in the vDA (Fig. 4H). Increased expression of GFP and *gfi1aa* mRNA within the inner ear in these embryos (Fig. 4F and H) reflects an expected increase in the number of *gfi1aa*-expressing hair cells in Notch-depleted embryos (Haddon et al., 1998).

Next, we examined whether qmc551: GFP induction in HECs was also dependent on Runx1. We found that the same *runx1* morphant embryos expressed GFP in the vDA (Fig. 4I), but displayed a loss of *cmyb* expression after fixation and WISH staining (Fig. 4J). Likewise, non-transgenic *runx1* morphants retained endogenous *gfi1aa* mRNA (Fig. 4K) while losing *cmyb* expression (data not shown). Midline *gfi1aa* expression had previously been shown to be independent of Runx1 and was, therefore, thought to be unrelated to definitive haematopoiesis (Cooney et al., 2013). Our transgenic line reveals that this Runx1-independent *gfi1aa* expression occurs in HECs. Interestingly, GFP expression in circulating prRBCs was reduced in *runx1* morphants, suggesting that *runx1* promotes *gfi1aa* expression in prRBCs (Fig. 4L).

### 2.5 The transposon interferes with normal *gfi1aa* transcription in primitive red blood cell progenitors, but erythrocyte differentiation is unaffected

**Fig. 5:**
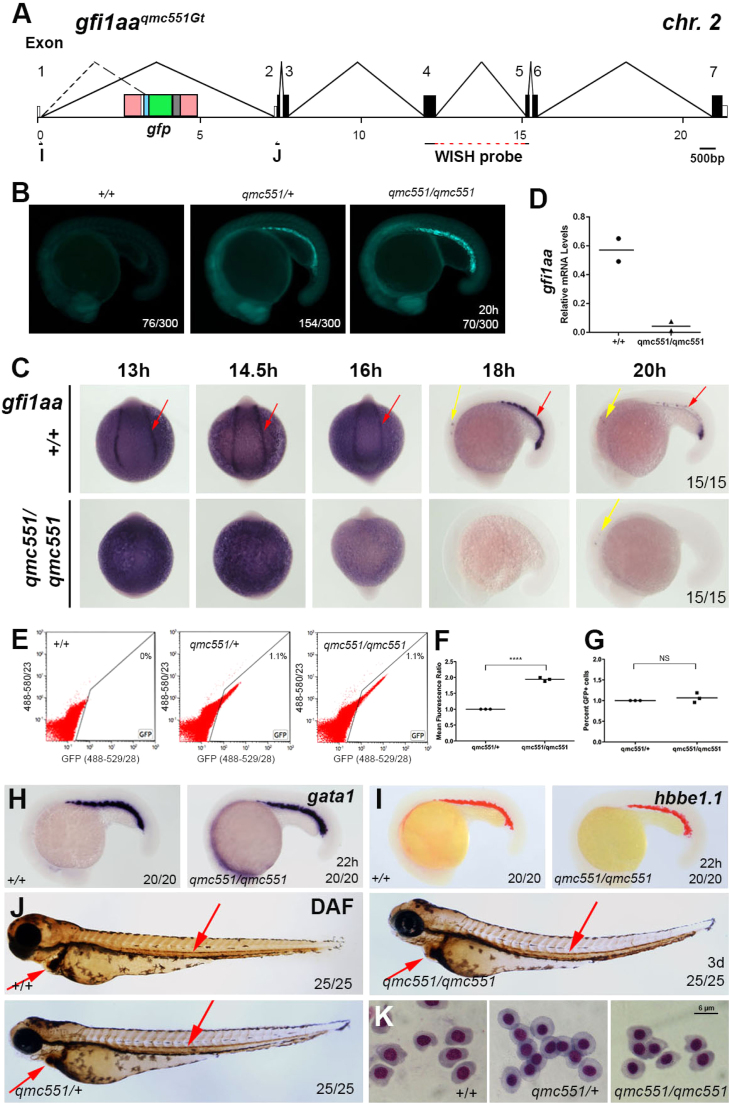

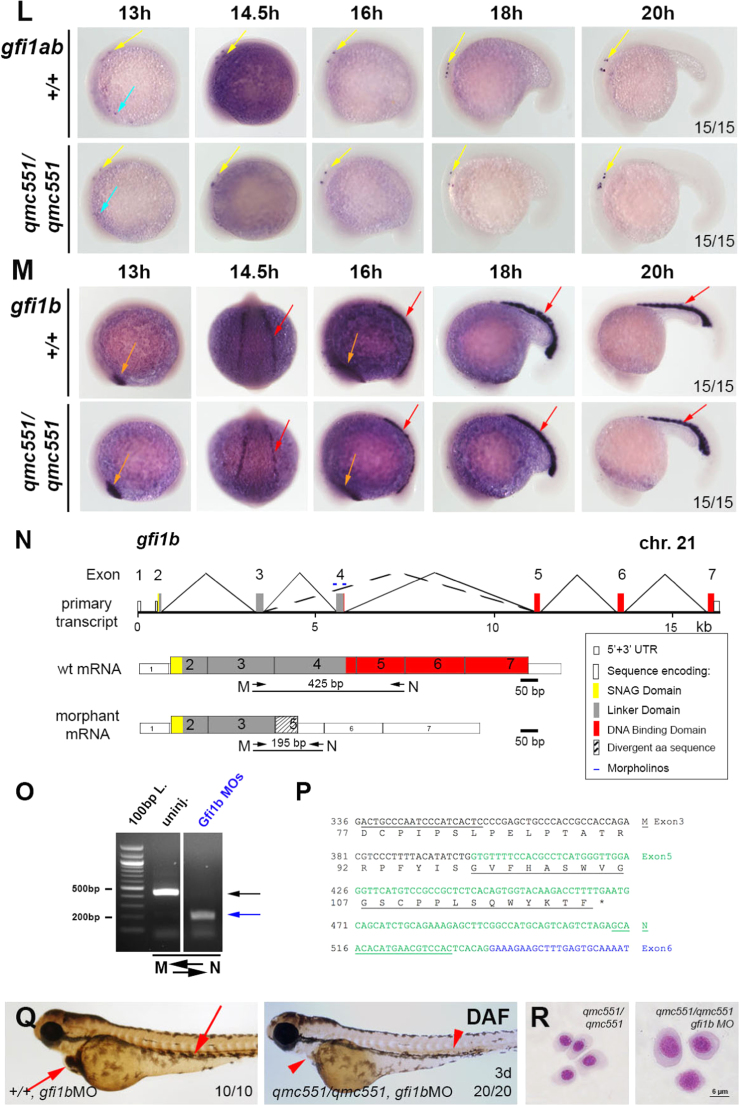
*Gfi1aa* expression is lost in primitive erythrocytes of homozygous *qmc551* embryos, yet primitive haematopoiesis is unaffected. (A) Genomic organization of the *gfi1aa*^*qmc551Gt*^ locus. Oligos I and J were used in the RT-PCR experiments. The sequence complementary to the *gfi1aa* WISH probe is indicated. The TaqMan probe overlapped with the exon 1/2 boundary and is not shown. **(B)** Fluorescent images of 20 hpf *wt*, *qmc551* heterozygous and homozygous siblings from an incross of *qmc551* heterozygous carriers. **(C,H,I,L,M)** RNA WISH experiments with indicated probes on 13–20 hpf *wt* and *qmc551* homozygous embryos. Views are posterior with anterior up on all images of 13–16 hpf and lateral with anterior to the left and dorsal up on all images of 18, 20 and 22 hpf embryos. **(D)** QRT-PCR to determine relative levels of *gfi1aa* mRNA in 16 hpf whole embryos. *EF1α* mRNA was used as a loading control. **(E)** Flow cytometric analysis of embryonic cells at 19 hpf showing green and red fluorescence excited with a 488 nm laser, and detected using the band pass filters 529/28 and 580/23 (central wavelength/width in nm), respectively. **(F)** Mean green fluorescence observed in GFP^+^ cells in embryos at 19 hpf. Please note that values are shown relative to heterozygous controls (Two-tailed *t*-test: p<0.0001). **(G)** The relative numbers of GFP^+^ cells in embryos at 19 hpf. **(J,Q)** Diaminofluorene staining to detect hemoglobin in 3 dpf embryos. **(K,R)** Images of prRBCs that were isolated from the sinus venosus of terminally anaesthetized 3 day-old embryos and stained with May-Grünwald and Giemsa. **(N)** Structure of the *gfi1b* transcript before and after splicing in the presence and absence of *gfi1b* morpholinos. In the morphant, exon 3 and 5 sequences are spliced together (dashed line). **(O)** RT-PCR performed on RNA isolated from uninjected and morpholino-injected *wt* embryos. **(P)***gfi1b* cDNA sequence representing the alternatively spliced *gfi1b* mRNA isolated from *gfi1b* morphant embryos. PCR oligo and divergent C-terminal amino acid sequences are underlined. Sequences derived from different exons are shown in different colors. The numbering of nucleotides corresponds to that of database entry NM_001271841. Encoded amino acids are counted below. Arrows: red – prRBCs, yellow – inner ear hair cells, light blue – anterior lateral mesoderm, orange – hatching gland precursors. Arrowheads: red – prRBCs in heart. Fractions x/y give the number of embryos, x, with depicted phenotype out of all embryos analyzed, y. Embryos are shown with anterior left and dorsal up.

The location of the gene trap transposon (Fig. 5A) suggested that it might interfere with the expression of the *gfi1aa* gene. To address this issue, *qmc551* heterozygotes and homozygotes were sorted based on the level of GFP fluorescence (Fig. 5B) and used in *gfi1aa* WISH. These experiments showed that *gfi1aa* expression was retained in the inner ear, but lost in the prRBCs of *qmc551* homozygotes (Fig. 5C). At 16 hpf, quantitative RT-PCR (qRT-PCR) confirmed that *gfi1aa* mRNA was substantially reduced in *qmc551* homozygotes (Fig. 5D). Despite this reduction, homozygous embryos did not appear to carry less prRBCs. Flow cytometric analyses revealed that the GFP^+^ *qmc551* homozygous prRBCs had a twofold higher mean fluorescence (Fig. 5E and F), but that prRBC numbers were similar in heterozygous and homozygous carriers (Fig. 5E and G). The normal number of prRBCs was also reflected in normal *gata1* and β-globin (*hbee1)* expression patterns in 22 hpf *qmc551* homozygotes (Fig. 5H and I). By day 3, circulating prRBCs of *qmc551* homozygotes stained normally with the hemoglobin peroxidase substrate diaminofluorene (DAF) (Fig. 5J) and displayed normal overall cell morphology (Fig. 5K), clearly demonstrating that prRBCs were not only specified, but also matured in the absence of Gfi1aa. Normal primitive erythropoiesis in *qmc551* homozygous embryos could be sustained due to functional redundancy between the three zebrafish Gfi1 paralogs. While *gfi1ab* expression could not be detected in prRBCs in the presence or absence of Gfi1aa (Fig. 5L), *gfi1b* expression was present and unaltered in the homozygous *qmc551* embryos (Fig. 5M). To test whether Gfi1b compensates for the loss of Gfi1aa expression during primitive erythropoiesis, Gfi1b morpholinos were injected into homozygous *qmc551* embryos. The morpholinos were designed to target the splice junctions that flank exon 4 of the primary *gfi1b* transcript (Fig. 5N). RT-PCR showed that injected morphant embryos carried an alternatively spliced *gfi1b* mRNA (Fig. 5O). The sequence of its RT-PCR fragment revealed that exons 3 and 5 were spliced together, leading to a shift of the *gfi1b* reading frame (Fig. 5N and P). This frame shift is predicted to cause the production of a truncated Gfi1b protein that retains the N-terminal 20 amino acid-long SNAG (SNAIL/GFI1) domain and parts of the linker domain, but lacks all Zn-fingers of Gfi1b's DNA binding domain. Instead, a divergent sequence of 22 amino acids forms the C-terminus of the truncated product. The loss of the DNA binding domain is likely to interfere with the protein's function. While injected *wt* embryos displayed normal erythropoiesis at 3 dpf, morphant *qmc551* homozygous embryos showed a dramatic reduction in DAF staining (Fig. 5Q). In comparison to the normal morphology of prRBCs in uninjected embryos, prRBCs of morphant *qmc551* homozygotes appeared larger and their nuclei were less condense (Fig. 5R). Nuclear condensation and a reduction in cell size are hallmarks of RBC differentiation (Qian et al., 2007, Weinstein et al., 1996). Thus, in the absence of Gfi1aa and Gfi1b, prRBCs failed to mature. The apparently normal maturation of prRBCs in uninjected *qmc551* homozygous embryos suggests that Gfi1b is sufficient to rescue primitive erythropoiesis in the absence of Gfi1aa.

### 2.6 Gfi1aa is not essential for definitive haematopoiesis

**Fig. 6:**
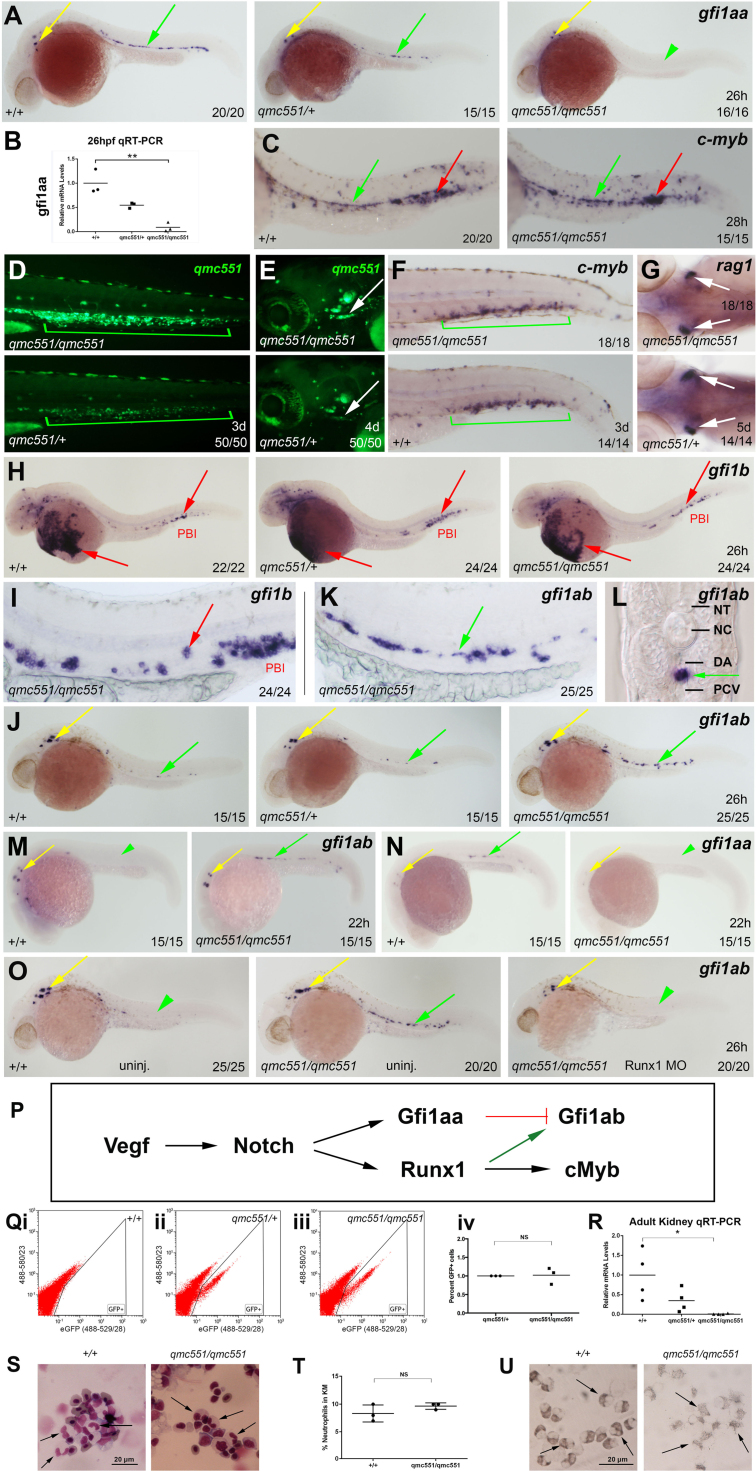
*Gfi1aa* expression is lost in definitive haematopoietic cells of homozygous *qmc551* fish, but definitive haematopoiesis is normal. **(A,C,F-O)** Fixed embryos stained in WISH experiments using indicated probes. Whole embryos are shown in (A,H,J,M-O). Close-up views of anterior and posterior parts of the embryos are presented in (G) and (C,F,I,K), respectively. All embryos, except (G), are shown in a lateral view with anterior to the left and dorsal up. (G) shows a close-up dorsal view with anterior to the left. (L) shows a transverse section through the trunk of one of the homozygous *qmc551* embryos after *gfi1ab* WISH. **(B,R)** QRT-PCR on total RNA isolated from whole 26 hpf embryos (B) and from adult KM cells (R) to measure the relative level of *gfi1aa* mRNA (Two-tailed *t*-test: p=0.002 (26 h), p=0.034 (KM)). *Ef1α* mRNA was used as a loading control. **(D,E)** Live images of *qmc551* embryos with anterior left and dorsal up. Close-up pictures of the tail (D) and the head regions (E) are presented. **(P)** Regulation of Gfi1aa and Gfi1ab expression at the onset of definitive haematopoiesis. **(Q)** Flow cytometric analysis of green and red fluorescence in adult KM cells excited with a 488 nm laser and detected using 529/28 and 580/23 nm band pass filters, respectively. Please note that a substantial proportion of KM cells displays green and red autofluorescence. **(S)** May-Grünwald/Giemsa stained KM cytospins. **(T)** Relative number of bilobed and trilobed neutrophil granulocytes observed in May-Grünwald/Giemsa stained cytospins. (U) Sudan Black staining on adult KM cytospins. Arrows: red – prRBCs, green – HECs and definitive HCs, yellow – inner ear hair cells, white – T cell precursors in the thymus, black – neutrophil granulocytes with bilobed nuclei. Arrowheads: green – reduced gene expression in the vDA.

Next, we examined whether the transposon also interfered with *gfi1aa* expression at the onset of definitive haematopoiesis. WISH experiments on 26 hpf *qmc551* homozygous embryos showed that *gfi1aa* expression was lost in the vDA, while expression was retained in the inner ear (Fig. 6A). QRT-PCR confirmed the substantial reduction in *gfi1aa* mRNA in the 26 hpf embryo (Fig. 6B). Despite this loss, *cmyb* expression in the vDA was normal (Fig. 6C) and GFP^+^ cells were observed to seed the CHT (Fig. 6D) and the thymus (Fig. 6E) of *qmc551* homozygous embryos. Their green fluorescence was matched by the presence of *cmyb*-positive HSPCs of the CHT and *rag1*-expressing T cell progenitors of the thymus (Fig. 6F and G), demonstrating that definitive haematopoiesis commenced as normal. Within the vDA, we found no evidence for *gfi1b* mRNA in *wt* or *qmc551* homozygous embryos (Fig. 6H and I). Gfi1b expression was clearly restricted to prRBCs in circulation over the yolk and in the PBI (Fig. 6H,I). By contrast, *gfi1ab* mRNA which had previously been shown to display a scattered expression pattern in the vDA (Dufourcq et al., 2004) was dramatically increased in the homozygous *qmc551* embryos (Fig. 6J). A close-up of the trunk region shows that the *gfi1ab* expression pattern displayed an almost continuous line in the embryonic midline (Fig. 6K). Transverse sections demonstrated that the staining was associated with the vDA (Fig. 6L), suggesting that *gfi1ab* expression was upregulated and likely to substitute for Gfi1aa at the onset of definitive haematopoiesis.

Upregulation of *gfi1ab* expression was first observed at 22 hpf (Fig. 6M). At this time, *gfi1aa* expression decreases in prRBCs and only individual cells located in the position of the future vDA display weak *gfi1aa* expression (Fig. 6N). Given that *gfi1ab* expression was not seen in prRBCs between 13 and 20 hpf in *wt* or *qmc551* homozygous embryos (Fig. 5L), the *gfi1ab*-positive cells seen at 22 hpf are most likely definitive precursor cells (Fig. 6M). Interestingly, when *gfi1ab* expression was examined in *qmc551* homozygotes that had been injected with a *runx1* morpholino, de-repressed *gfi1ab* expression was completely lost at 26 hpf (Fig. 6O), suggesting that, unlike *gfi1aa*, *gfi1ab* expression at the onset of definitive haematopoiesis requires direct or indirect activation by Runx1 (Fig. 6P).

Homozygous *qmc551* larvae were successfully raised to adulthood. Adults were perfectly viable and fertile, and showed no phenotypic abnormalities. Flow cytometric analysis of adult KM cells showed that the relative numbers of GFP^+^ cells were identical in *qmc551* heterozygous and homozygous adult fish (Fig. 6Q), despite a substantial reduction in *gfi1aa* mRNA in KM cells of *qmc551* homozygotes (Fig. 6R). As Gfi1 knockout mice display neutropenia, KM cytospins were prepared to examine neutrophil granulocytes in *qmc551* homozygous fish. KM cytospins stained with May-Grünwald and Giemsa revealed cells with bilobed and trilobed nuclei (Fig. 6S), which represented about 10% of the KM cells in *wt* and *qmc551* homozygotes (Fig. 6T). These cells could successfully be stained with the neutrophil granulocyte stain Sudan Black (Fig. 6U). Thus, *qmc551* homozygous fish displayed no obvious signs of neutropenia. Altogether, our data showed that although the gene trap transposon abrogates *gfi1aa* expression in haematopoietic cells, primitive and definitive haematopoiesis remain unaffected.

## 3 Discussion

The zebrafish line *qmc551* carries a GFP gene trap transposon within intron 1 of the gene *gfi1aa* on chromosome 2. RT-PCR confirmed the presence of a spliced mRNA that fuses *gfi1aa* 5′UTR sequences encoded by exon 1 to the splice acceptor on the gene trap, showing that GFP transcription is under the control of the *gfi1aa* promoter. GFP translation is ensured by an ATG start codon at the beginning of the *gfp* open reading frame. The GFP reporter faithfully recapitulates early embryonic *gfi1aa* expression inside and outside the haematopoietic system. Furthermore, it provides a sensitive live read-out of *gfi1aa* promoter activity in cell types and at times when *gfi1aa* promoter activity cannot easily be detected by WISH. The expression pattern of qmc551: GFP is much wider than that of the previously published *gfi1aa* enhancer trap line *gfi1.1: gfp* whose GFP expression was limited to prRBCs (Wei et al., 2008). The *gfi1.1: gfp* line's enhancer trap was inserted 20 kb upstream of *gfi1aa,* and its GFP expression was probably dependent on a single local enhancer. By contrast, our gene trap's GFP reporter is likely to be under the control of all cis-regulatory elements that regulate the activity of the endogenous *gfi1aa* promoter upstream of exon 1.

Our WISH and qRT-PCR data showed that the gene trap transposon interferes with *gfi1aa* transcription in pRBCs, in HECs of the vDA and in adult KM cells. The absence of exon 4/5-containing RNA sequences suggests that the primary transcript is terminated at the SV40 polyadenylation signal downstream of the *gfp* reading frame. Our WISH data also revealed that the transposon-mediated suppression of *gfi1aa* transcription is context-dependent. Hair cells in the inner ear express GFP, but also retain *gfi1aa* expression. The remaining expression may be due to inefficient transcript termination or the use of an alternative promoter downstream of the transposon. It is noteworthy that a transcript initiated in intron 1 would encode a full-length Gfi1aa protein. Interestingly, analysis of genome-wide data on histone 3 lysine 4 trimethylation, an epigenetic mark enriched at transcriptionally active promoters, in 24 hpf zebrafish embryos (Aday et al., 2011) shows that *gfi1aa*'s intron 1 sequences are associated with this mark. The lack of inner ear defects in *qmc551* homozygous embryos is likely due to the residual Gfi1aa expression and the co-expression of Gfi1ab in the sensory hair cells. This example highlights that one cannot simply assume that the transposon interferes with *gfi1aa* expression in all cell types and at all differentiation stages. A more detailed analysis into this context dependency was outside the scope of this study.

Despite the loss of Gfi1aa expression in prRBCs, homozygous *qmc551* embryos displayed normal primitive erythropoiesis. PrRBC progenitors were specified in normal numbers and DAF staining suggested that they matured normally. These finding contradict previous morpholino studies that proposed an essential role for Gfi1aa in primitive erythropoiesis (Cooney et al., 2013, Wei et al., 2008). The convincing reduction in *gfi1aa* mRNA in our mutants suggests that the reported morphant phenotype is possibly due to off-target effects (Lawson, 2016, Stainier et al., 2015). The normal development of prRBCs in our mutants is consistent with the phenotype of the mouse Gfi1 knockout. As in zebrafish, mouse prRBC progenitors co-express Gfi1 and Gfi1b (Moignard et al., 2015), and single *Gfi1* and *Gfi1b* knockout mice display normal primitive erythropoiesis (Hock et al., 2003, Saleque et al., 2002). By contrast, loss of both proteins causes reduced embryonic βH1 globin expression in the murine yolk sac, suggesting a defect in primitive erythropoiesis (Lancrin et al., 2012). We show here that morpholino-mediated knockdown of Gfi1b in Gfi1aa-deficient embryos also interfered with primitive erythropoiesis in zebrafish. Initially, prRBCs were specified, but subsequently failed to mature. The lack of a maturation defect in either *gfi1aa* mutant or *gfi1b* morphant embryos demonstrated that Gfi1aa and Gfi1b could substitute for each other during primitive erythropoiesis in zebrafish.

**Fig. 7:**
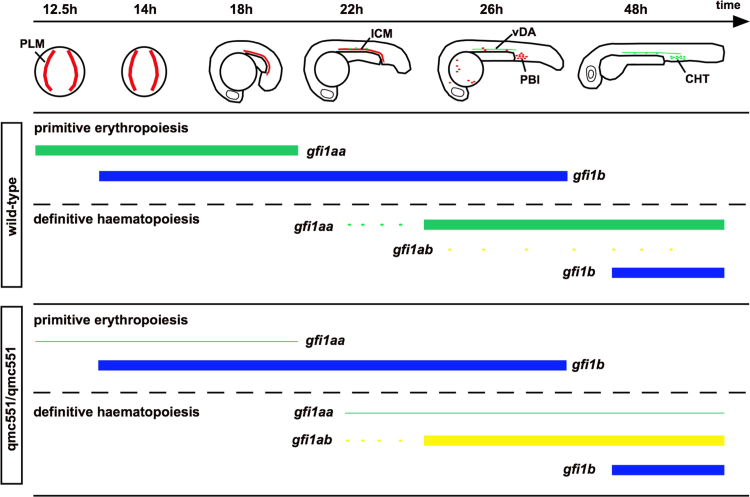
Expression of zebrafish *gfi1/1b* paralogs during primitive erythropoiesis and definitive haematopoiesis in wild-type and homozygous *qmc551* embryos. Top – Diagrammatic representation of zebrafish embryos from 12.5 to 48 hpf. The images show posterior and lateral views of early and late embryos, respectively. Sites of primitive erythropoiesis (in red) and definitive haematopoiesis (in green) are depicted. Abbreviations: PLM – posterior lateral mesoderm; ICM – intermediate cell mass; PBI – posterior blood island; CHT – caudal haematopoietic tissue. Bottom – Expression of *gfi1* paralogs in *wt* and *qmc551* homozygous embryos. Solid boxes represent strong expression. Dots show scattered expression. Lines represent apparent loss of expression.

Homozygous *qmc551* carriers were viable and fertile, and displayed none of the phenotypic abnormalities observed in the definitive blood system of Gfi1 knockout and Gfi1: GFP knock-in mice (Hock et al., 2003, Karsunky et al., 2002, Wallis et al., 2003, Yücel et al., 2004). In particular, the neutrophil granulocytes, which are severely reduced in Gfi1-depleted mice, were present in normal numbers. The normal blood phenotype was likely due to functional redundancy with Gfi1aa's paralogs (Fig. 7). At the onset of definitive haematopoiesis, loss of Gfi1aa expression lifted the repression of *gfi1ab* and led to the Runx1-dependent upregulation of Gfi1ab in HECs of the DA. Mammalian Gfi1 and Gfi1b proteins are known to auto- and cross-regulate their expression in a context-dependent manner (Doan et al., 2004, Montoya-Durango et al., 2008, Yücel et al., 2004). In homozygous *qmc551* embryos, upregulation of *gfi1ab* was first detected in individual cells of the ICM. Based on their position within the ICM and the lack of *gfi1ab* upregulation in prRBCs at earlier time points, we suppose that these cells represent progenitors of HECs, i.e. haemogenic aortic angioblasts. These cells may be equivalent to the suspected HSC precursors recently reported to express *gata2b* (Butko et al., 2015), an issue that requires further attention.

In the mouse, loss of Gfi1 alone does not abrogate EHT. Only *Gfi1/Gfi1b* double knockout mice display deficiencies in HECs of the YS and the vDA. In the YS, HEC-derived HCs cannot down-regulate EC genes and fail to enter circulation (Lancrin et al., 2012). In the vDA, HECs are specified, but fail to undergo EHT (Thambyrajah et al., 2015). In zebrafish, we did not see any convincing *gfi1b* expression in vDA HECs, but *gfi1b* expression was observed in definitive HCs of the CHT. Whether it plays an important role in EHT remains to be determined. In addition, the strong expression of *gfi1aa* in *wt* and *gfi1ab* in Gfi1aa-deficient embryos casts doubts on the previous morpholino-based assumption that Gfi1b alone is essential for the formation of all definitive haematopoietic lineages (Cooney et al., 2013). In the mouse, Gfi1 and Gfi1b are co-expressed not only during EHT, but also in HSCs, and only the loss of both genes completely abrogates HSC maintenance (Hock et al., 2004, Khandanpour et al., 2010, Zeng et al., 2004). Single *Gfi1b* knockout mice display severe defects only in cell types that do not co-express Gfi1, i.e. definitive erythrocytes and megakaryocytes (Saleque et al., 2002). The generation of *gfi1ab* and *gfi1b* single, as well as double and triple mutants in zebrafish is needed to shed more light on the redundant and non-redundant roles of these proteins during definitive haematopoiesis. The lines will also allow us to carefully examine dose-dependent requirements for Gfi1/1b proteins during tissue differentiation. Unlike the previous morpholino results, our data on the *qmc551* line are consistent with findings in the mouse and strongly support the notion that the roles of Gfi1 and Gfi1b are at large conserved between teleosts and mammals. The high level of GFP expression in our *qmc551* line and the transparency of the zebrafish embryo will be instrumental in unraveling the behavior of cells in *gfi1aa/gfi1ab/gfi1b* and other mutant backgrounds. This line will also be an excellent resource for HECs and HSPCs for use in transplantation and biochemical characterization.

## 4 Materials and methods

### 4.1 Zebrafish husbandry and experimentation

Information on zebrafish husbandry is provided in Supplementary data. Genetically altered zebrafish are listed in Table S1.

### 4.2 Transgene construction and molecular biology experiments

Details on the transposon Tol2-embedded gene trap, the *flk1: tdTom* and the *csl: cer* constructs are available upon request. To generate transgenic lines, plasmid constructs were injected with Tol2 transposase mRNA into one-cell stage embryos (Kotani et al., 2006). Embryos that harbored cells with transient reporter expression that contributed normally to embryonic development were raised. Adults were crossed to *wt* fish to identify transgenic founders. Their progeny established the lines reported herein. Southern Blots followed standard procedures. The integration site in *qmc551* was identified via inverse PCR as previously described (Kotani et al., 2006). Briefly, genomic DNA from *qmc551* embryos was digested with *MboI*, self-ligated and used in a nested PCR. The amplification product was cloned and sequenced. A Blast search in ENSEMBL (Flicek et al., 2014) on zebrafish genome assembly zv8 allowed the localization of the transposon. Total RNA was isolated from *qmc551* embryos using the RNeasy mini Kit (Qiagen) and was reverse transcribed using Superscript II reverse transcriptase (Life Technologies). Standard PCRs on cDNA and genomic DNA were performed using Taq polymerase (New England Biolabs). Quantitative TaqMan PCR on cDNA employed the qPCR mix plus Rox reference dye (Thermo Scientific). Oligo sequences are provided in Table S2. Statistical analyses were performed using Graph Pad Prism.

### 4.3 RNA in situ hybridization and immunohistochemistry

Alkaline phosphatase and tyramide fluorescent WISH experiments were performed using published protocols (Broadbent and Read, 1999, Schoenebeck et al., 2007). Immunodetection followed standard protocols, using reagents summarized in Table S3. Some stained embryos were embedded in JB4 methacrylate (Agar Scientific, Cambridge) and sectioned on a Leica RM2265 microtome. Sudan Black staining of embryos followed (Le Guyader et al., 2008). Kidneys were isolated (Gerlach et al., 2011), fixed in 4% paraformaldehyde overnight, soaked in 30% sucrose overnight, frozen in OCT and sectioned on a Leica CM1850 cryostat.

### 4.4 Morpholino injections and inhibitor treatments

Morpholinos (see Table S2) were injected in a volume of 0.5 nl into 2–4 cell stage embryos. The two *gfi1b* morpholinos (0.5 ng each) were co-injected with 0.5 ng of p53 morpholino. The latter was used to block the frequently observed morpholino-induced upregulation of p53 and the p53-induced apoptosis (Robu et al., 2007). Inhibitors were added to the embryo medium and applied from tailbud stage. Control embryos were treated with the solvent DMSO. The Diaminofluorene staining followed (Weinstein et al., 1996). To block pigmentation and immobilize live embryos for confocal imaging, embryos were treated with MS222, and phenylthiourea, and embedded in 1% low melting point agarose as described in (Renaud et al., 2011). Details on chemicals are given in Table S3.

### 4.5 Fluorescence-activated cell sorting and cytospins

Blood cells were collected from adult kidneys as described (Traver et al., 2003). Forward scatter, side scatter and GFP/dsRed fluorescence characteristics of KM cells were analyzed on a Beckman Coulter MoFlo Astrios cell sorter using the Kaluza software. Sytox was used to exclude dead cells. Using a Shandon Cytospin 4, all or just the GFP^+^ KM cells were cytocentrifuged for 3 min onto slides at 300 rpm and medium acceleration. RBCs were isolated from the sinus venosus of terminally anaesthetized 3 day-old embryos. The cells were subsequently stained with May-Grünwald, Giemsa or Sudan Black following manufacturer's instructions (see Table S3).

### 4.6 Microscopy and Imaging

Embryos were examined on a Nikon SMZ-1500 microscope. Sections and cytospins were analyzed on a Nikon Eclipse i80. Images were taken with a Nikon DS-5Mc/DS-U1 camera setup operated by the Nikon ACT-2U software or captured with a monochrome Hamamatsu Orca-ER camera via IP Lab software. Orca black and white images were pseudo-colored. Confocal microscopy was performed on Zeiss Exciter, LSM510 and LSM710 inverted confocal microscopes via ZEN software. All confocal images were analyzed in Imaris (Bitplane). Videos exported from Imaris were annotated in iMovie. Images were collated in Photoshop CS6.

## Competing interests

No competing interests declared.

## Author contributions

RT identified the *qmc551* line and performed its initial characterization. DU revealed that haematopoietic *gfi1aa* expression is lost in homozygous carriers, and studied haematopoiesis in the absence of Gfi1aa. MJ performed all confocal timelapse analyses and immunohistochemistry on kidney sections. DU and YH performed the *gfi1b* morpholino experiments. RNW helped evaluate data and examined possible expression of qmc551: GFP in macrophages. KM and CM helped PhD students in experimental design and data analysis. MG supervised the project and wrote the manuscript.

## Funding

This work was supported by Medical Research Council grants [79780 and MR/J000841/1 to MG], a BBSRC PhD studentship to RT (student number: 4055674), an MRC studentship to YH (student number: 4254522), and University of Nottingham International PhD scholarships to DU (student number: 4154910) and MJ (student number: 4191879).

## Footnotes

Appendix A Supplementary data associated with this article can be found in the online version at 10.1016/j.ydbio.2016.07.010.
