# Supplementary material for: A gene trap transposon eliminates haematopoietic expression of zebrafish Gfi1aa, but does not interfere with haematopoiesis
Source: Dev Biol. 2016 Sep 1;417(1):25–39. doi: 10.1016/j.ydbio.2016.07.010 (PMC5003831; doi:10.1016/j.ydbio.2016.07.010)
Supplement: Supplementary file 7 — Supplementary material [file mmc1.docx]

**Supplementary Materials and Methods**

**Zebrafish husbandry and genetically altered zebrafish lines**

Zebrafish were kept at 28.5˚C as described in the zebrafish book (Westerfield, 2000). All experiments performed on animals were approved by the local ethical review committee and performed under the Home Office project licenses 40/2983 and 40/3457. All genetically altered lines are described in Table S1.

**Table S1: Zebrafish lines used in this study.**

| **Name of line** | **Abbreviation** | **Reference** |
| --- | --- | --- |
| *gfi1aa^qmc551Gt^* | *qmc551* | this manuscript |
| Tg(*flk1/kdrl:tdTomato*)^qmc64^ | *flk1:tdTom* | this manuscript |
| Tg(*csl:cerulean*)^qmc63^ | *csl:cer* | this manuscript; derivative of *csl:venus* (Gray et al., 2007) |
| Tg(*gata1:dsRed*)^ds2^ | *gata1:dsRed* | (Traver et al., 2003) |
| Tg(*flk1/kdrl:egfp*)^s843^ | *flk1:gfp* | (Jin et al., 2005) |
| Tg(*mpeg1:mCherry*)^gl22^ | *mpeg1:mCherry* | (Ellett et al., 2011) |
| *mib^ta52b^* | *mib* | (Itoh et al., 2003) |

**Table S2: Oligonucleotides and morpholinos.**

| **Experiment** | **Oligo Name** | **Sequence** |
| --- | --- | --- |
| inverse PCR in Fig S1 | f1 | 5’-AGTACTTTTTACTCCTTACAATT-3’ |
| inverse PCR in Fig S1 | r1 | 5’-GATTTTTAATTGTACTCAAGTAA-3’ |
| inverse PCR in Fig S1 | f2 | 5‘-GGAGATCACTTCATTCTATTTTCC-3’ |
| inverse PCR in Fig S1 | r2 | 5’-CAAGTAAAGTAAAAATCCCCAA-3’ |
| PCR in Fig. 2D,E | A | 5’-CGGAGGAACTGTTCACCTACAG-3’ |
| PCR in Fig. 2D,E | B | 5’-CCATGCCGAGAGTGATCCCG-3’ |
| PCR in Fig. 2D,E | C | 5’-GGACGGCGACGTAAACGGC-3‘ |
| PCR in Fig. 2D,E | D | 5’-CTACCTGGGTCTCCACTTTTGC-3’ |
| PCR in Fig. 2D,F | E | 5’-CCACAAGTTCAGCGTGTCC-3’ |
| PCR in Fig. 2D,F | F | 5’-AACTCCAGCAGGACCATGTG-3’ |
| RT-PCR in Fig. 2D,G | G | 5’-CTCTATAGCGGGACACAGGAG-3’ |
| RT-PCR in Fig. 2D,G | H | 5’-GGACACGCTGAACTTGTGG-3’ |
| gfi1aa qRT-PCR in Fig. 5+6 | I | 5’-CAGAGCAGCACAGCATTA-3’ |
| gfi1aa qRT-PCR in Fig. 5+6 | J | 5’-ACTGCCCAATCCCATCACTC-3’ |
| gfi1aa qRT-PCR in Fig. 5+6 | gfi1aa probe | 5’-ACGTCACCATCATTGATGCCCCTGGA-3’ |
| ef1a qRT-PCR in Fig. 5+6 | K | 5’-TGGAAATTCGAGACCAGCAAA-3’ |
| ef1a qRT-PCR in Fig. 5+6 | L | 5’-AGTCAGCCTGAGAAGTACCAGTGA-3’ |
| ef1a qRT-PCR in Fig. 5+6 | ef1a probe | 5’-AGCTCTCCAATCTAGCCTGTTGCTGT-3’ |
| gfi1b RT-PCR oligo in Fig. 5 | M | 5’-ACTGCCCAATCCCATCACTC-3’ |
| gfi1b RT-PCR oligo in Fig. 5 | N | 5’-GTGGACGTTCATGTGTTGCT-3’ |
| Splice morpholino against runx1 transcript | runx1 MO | 5’ AGCGCTCTTACCGTATTTGTCC-3’ (Gering and Patient, 2005) |
| Splice morpholino against *rbpja/b* transcript | rbpja/b MO | 5’-CAAACTTCCCTGTCACAACAGGCGC-3’ (Sieger et al., 2003) |
| Splice morpholino 1 against *gfi1b* | MO1 | 5’ –TCTGATGGAGGTATGATGGAAACAT-3’ |
| Splice morpholino 2 against *gfi1b* | MO2 | 5’-GCTGTGTTCACTATCTGACCTTGTC-3’ |
| p53 morpholino | p53 MO | 5’-GCGCCATTGCTTTGCAAGAATTG-3’ {Robu:2007ed} |

**Table S3: Information on antibodies, selected chemicals and inhibitors**

| **Chemical/Inhibitor** | **Company** | **Cat. No.** | **Instructions** |
| --- | --- | --- | --- |
| Biotin-conjugated goat anti-GFP antibody | Abcam | Ab6658 | Use 1:250 diluted |
| Steptavidin-Alexa Fluor 488 | Invitrogen | S32354 | Use 1:400 diluted |
| ABC Kit | Vector | PK-6100 | Follow manufacturer’s instructions |
| Diaminobenzidine | Sigma-Aldrich | D3939 | Follow manufacturer’s instructions |
| Rabbit anti-dsRed antibody | Clontech | 632496 | Use 1:250 diluted |
| Anti-rabbit IgG Alexa Fluor-594 | Invitrogen | A11037 | Use 1:400 diluted |
| 2,7-Diaminofluorene | Sigma-Aldrich | D17106 | Follow (Weinstein et al., 1996) |
| JB4 methacrylate | Agar Scientific UK | AGR1130 | Follow manufacturer’s instructions |
| OCT | VWR | 361603E | Follow manufacturer’s instructions |
| VEGFR kinase inhibitor | Calbiochem | 676475 (no longer available) | Stock at 10 mM in DMSO; use at 2.5 μM |
| SU5416 | Calbiochem | 676487 | Stock 10 mM in DMSO; use at 2.5 μM |
| DAPM | Calbiochem | 565777 | Stock at 12.5 mM in DMSO; use at 100 μM |
| MS222 | Sigma-Aldrich | A5040; | Stock at 4 g/l H_2_O, pH7.5; use at 130 μg/ml |
| N-Phenylthiourea | Sigma-Aldrich | P7629 | Stock at 0.3g/l H_2_O, use at 0.03 g/l |
| May-Grünwald | Sigma-Aldrich | MG1L-1L | Use undiluted |
| Giemsa | BDH | 350864X | Use 1:20 diluted |
| Sudan Black | Sigma-Aldrich | 380B-1KT | Follow manufacturer’s instructions without counterstain |
| DPX Mountant for Histology | Fluka BioChemika | 44581 | Apply directly to dried slide and put cover slip on top |

**Supplementary Figures**

**
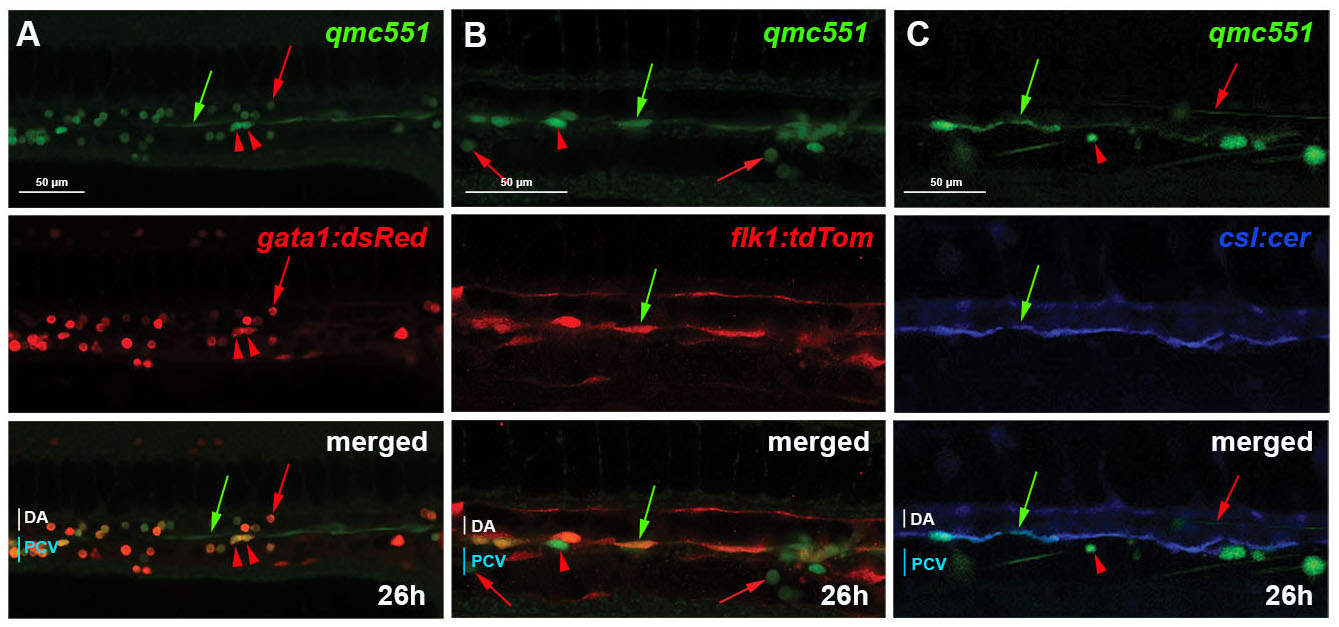
**

**Figure S1: In the gene trap line *qmc551*, GFP is expressed in primitive erythrocytes and in endothelial cells of the ventral wall of the dorsal aorta.** This figure supports data shown in Fig. 1. **(A)** Single color and merged confocal images of the trunk of a *qmc551;gata1:dsRed* double transgenic embryo after fluorescent GFP and dsRed immunostaining. **(B)** Single color and merged confocal images of a *qmc551;flk1:tdTom* double transgenic embryo after fluorescent immunostaining for GFP and tdTom. **(C)** Single color and merged confocal images of a live *qmc551*;*csl:cer* double transgenic embryo. Note that prRBCs in circulation appear as short lines in the confocal image in (C), while stationary cells are round. Images were taken at single cell resolution on 2 (A), 1.8 (B) and 2.5 (C) μm optical slices. Times given on the panels represent hours and minutes after fertilization. All images show embryos with anterior to the left and dorsal up. Red arrows - prRBCs; red arrowheads - prRBC progenitors trapped in the mesenchyme; green arrows - ECs in the vDA.

**
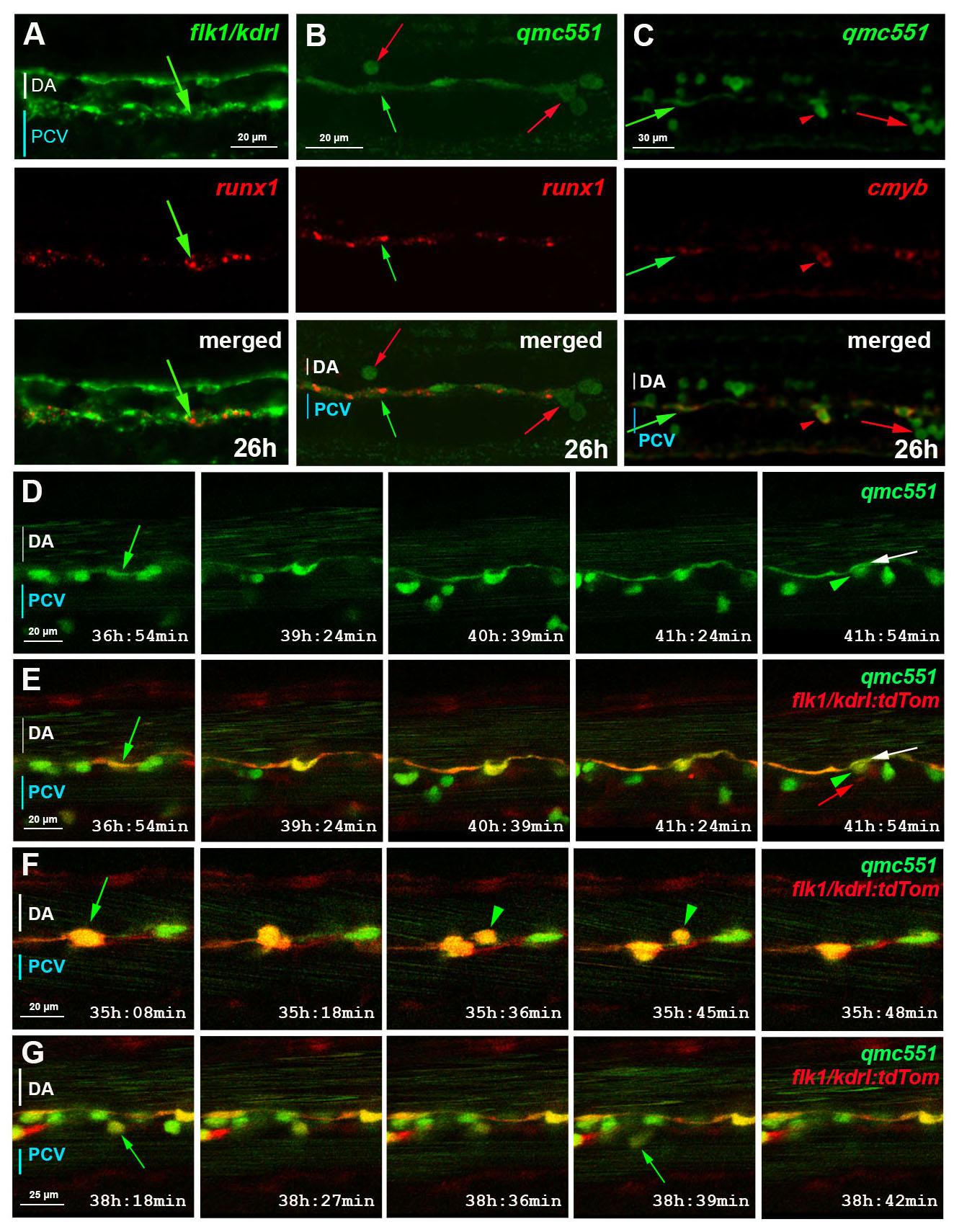
**

**Figure S2. qmc551:GFP positive endothelial cells are haemogenic endothelial cells.** This supplementary figure is related to Fig. 1. Confocal images of fixed (A-C) and live (D-G) embryos show optical sagittal sections through the DA with anterior left and dorsal up. The optical slices were at single cell resolution thickness, i.e. 2.0 (A,C), 1.0 (B) and 2.1 μm (D-G). Images in (D-G) are taken from timelapse experiments in which pictures were taken every 3 minutes. Times on panels represent hours and minutes after fertilization. **(A)** Double fluorescent *runx1* and *flk1/kdrl* whole-mount in situ hybridisation (WISH). **(B-C)** Fluorescent *runx1* (B) and *cmyb* (C) WISH combined with GFP immunohistochemistry. prRBCs (red arrows); prRBC progenitors in mesenchyme (red arrowheads) and HECs (green arrows). **(D-G)** Timelapse microscopy on *qmc551;flk1:tdTom* embryos from 31 hpf. (D) shows GFP expression only, while (E-G) present merged images. Annotations: HECs before (green arrow) and after bEMT (green arrowhead); DA endothelium after bEMT of HEC (white arrow); venous EC (red arrow).

**
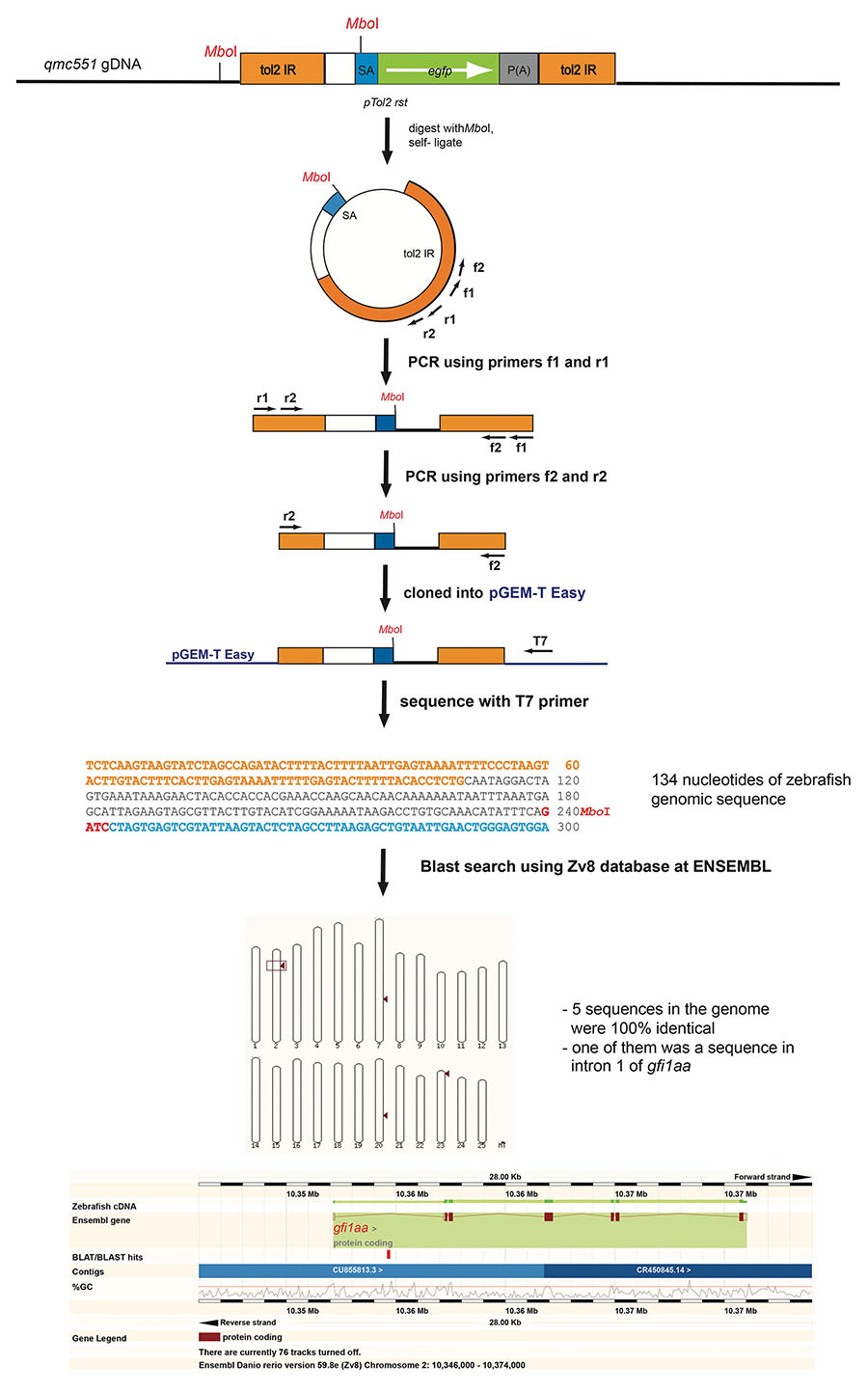
**

**Figure S3: The gene *gfi1aa* on chromosome 2 was identified as the gene trapped in the gene trap line *qmc551*.** This supplementary figure is related to Fig. 2. The diagram depicts the nested inverse PCR strategy that was used to clone the DNA upstream of the transposon from genomic DNA of *qmc551* embryos. The genomic DNA was digested with *MboI*. Individual fragments were self-ligated at low concentration using T4 DNA ligase. Nested PCR was performed using primers f1 and r1 in PCR1 and f2 and r2 in subsequent PCR2. The product of the second PCR was cloned into a pGEM-T Easy vector. The inserted DNA was sequenced using a T7 primer. Sequencing revealed the 134 bp sequence located upstream of the transposon. A BLAST search of the Zv8 assembly of the zebrafish genome in the Ensembl database (Flicek et al., 2014) revealed 5 hits. One of these hits was a sequence in intron 1 of *gfi1aa* on chromosome 2.


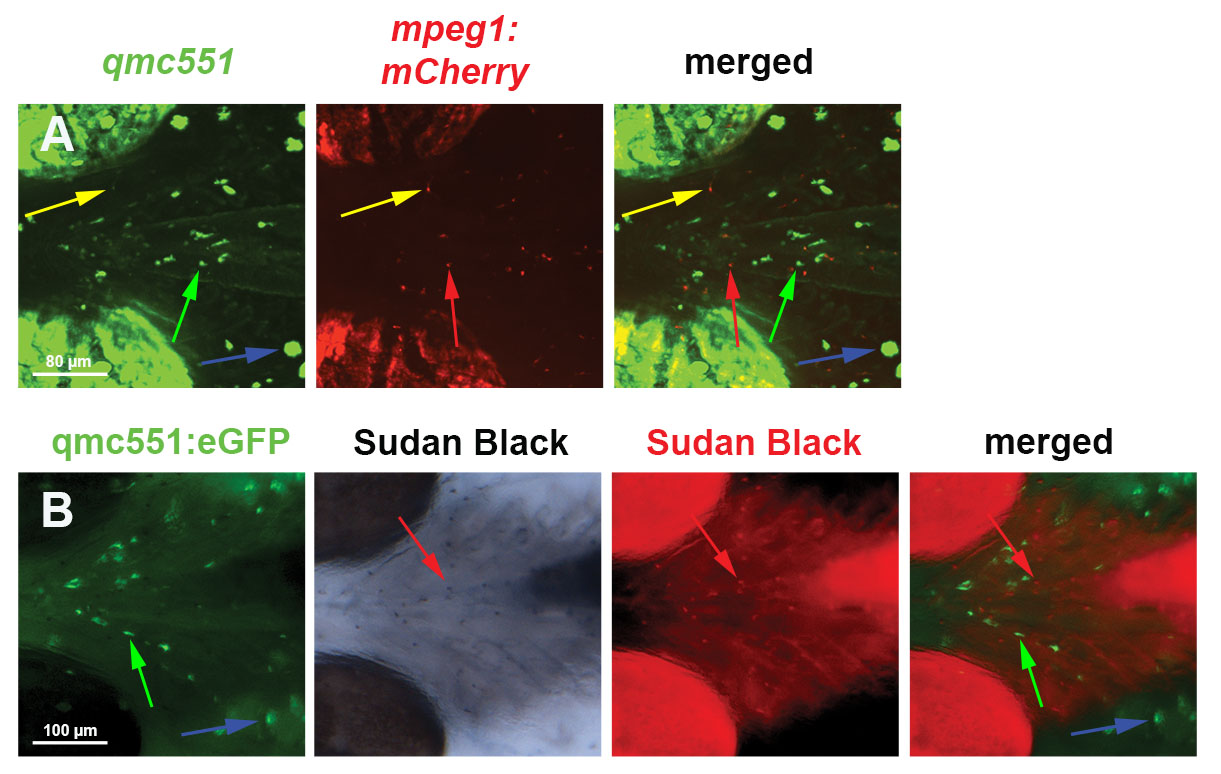


**Figure S4: Most GFP-positive cells that patrol the head tissues of *qmc551* transgenic embryos do not express markers of mature macrophages and neutrophil granulocytes.** (A) Live embryos that carry the *qmc551* gene trap and the macrophage reporter transgene *mpeg1:mCherry* display numerous GFP (green arrow) and mCherry (red arrow) single-positive cells, but hardly any double-positive cells (yellow arrow). (B) Fixed *qmc551* transgenic embryos stained for the mature neutrophil marker Sudan Black and immunostained for GFP harbor many cells that are single-positive for GFP (green arrow) and for Sudan Black (black in the original image; red in the pseudocolored image; red arrow). All images show ventral views of the head region of the embryo, with anterior to the left.

**Movie Legends**

**Movie 1: In the gene trap line *qmc551*, GFP is expressed in primitive red blood cells and in spindle-shaped cells located between the dorsal aorta and the posterior cardinal vein at 26 hpf.** This movie is related to Figure 1. **(A)** Live movie of the trunk and tail of a 26 hpf *qmc551* transgenic. GFP is expressed in circulating and stationary blood cells and in elongated cells located between the dorsal aorta and the posterior cardinal vein. Anterior is to the left, dorsal is up. Pictures of this timelapse movie were taken every 100 ms with a Hamamatsu Orca-ER camera on a Nikon SMZ1500 dissection microscope with an epifluorescence attachment using a FITC filter set. The camera was controlled by IP lab software. Images were pseudo-colored in IP lab and saved as TIFF files. The series of TIFF files was imported into Imaris and turned into a video that was then annotated in iMovie. **(B)** Confocal analysis of the trunk of a fixed 26 hpf *qmc551* transgenic embryo shows expression of GFP in blood cells and in spindle-shaped cells located between the lumen of the dorsal aorta and the lumen of the vein. An animation of a 3D maximum intensity projection of a 67.5 μm thick Z stack is shown. A still image of the maximum intensity projection of the Z stack highlights GFP+ endothelial cells (green arrows), red blood cells in circulation (red arrow) and outside the vessels (red arrowheads). The embryo was first treated with an overdose of anesthetic and then fixed in 4% PFA for an hour. The Z-stack was taken on an inverted Zeiss Exciter confocal microscope and has the dimensions 161 x 161 x 67.5 μm, respectively. The movie starts with anterior to the left and dorsal up. The confocal images were acquired on a Zeiss Exciter microscope with an EC Plan-Neofluar 40x/1.30 Oil DIC M27 lens.

**Movie 2: qmc551:GFP+ haemogenic endothelial cells undergo basal epithelial to mesenchymal transition.** This movie is related to Figure 1. It shows image series and still frames of confocal timelapse microscopy experiments performed on *qmc551:eGFP;flk1/kdrl:tdTom*-double transgenic embryos. The embryo was immobilized in 1% low melting point agarose. The embryo faces to the left with dorsal up. The timelapse experiment started at 31 hpf. In (A) only GFP expression is shown, highlighting that qmc551:GFP marks HECs before, during and after epithelial to mesenchymal transition. In (B), both GFP and tdTom fluorescence are shown. tdTom expression confirms that spindle-shaped GFP+ cells undergoing EMT are vDA ECs. tdTom also highlights the outline of the vein. Please note that green horizontal lines in the DA and the PCV are caused by GFP+ prRBCs in circulation. The still frame shows the final image of the timelapse series shown in (B). It highlights the HEC-derived mesenchymal cell (yellow arrow) and the endothelium (white arrow) that has closed up above it. All images show single 2.1 μm optical sections. Images were processed in Imaris and annotated in iMovie.

**Movie 3: qmc551:GFP+ haemogenic endothelial cells give rise to blood cells that join the circulation.** This movie relates to Figure 1. **(A)** Timelapse movie of 1.5 μm thick optical slices through the trunk of uninjected *qmc551;flk1/kdrl:tdTom* double transgenic embryos from 33 hpf. The movie shows how a double-positive haemogenic endothelial cell leaves the endothelium apically to enter the DA. **(B)** Mesenchymal GFP single (green circle) and GFP/tdTom double-positive (yellow circle) cells migrate through the mesenchyme before they enter the vein to join the circulation. This movie is identical to the one shown in Movie 2, but different cells are highlighted. The cell marked with the yellow circle disappears in Z as it moves from below the vein to a position above the vein. It then re-appears (yellow arrow) before it enters the vein. All images show single 2.1 μm optical sections. Images were processed in Imaris and annotated in iMovie.

**Movie 4: flk1/kdrl:GFP/csl:Cer-double positive haemogenic endothelial cells give rise to blood cells that join the circulation.** This movie provides supporting evidence related to Figure 1. It shows data from timelapse confocal microscopy on *flk1/kdrl:gfp/csl:cer*-double transgenic embryos starting from 48 hpf. Images of single 1.5 μm optical sections are shown with anterior to the left and dorsal up. Images were taken every 3 minutes. At 48 hpf, endothelial cells of the dorsal aorta co-express both reporter transgenes, while vein endothelial cells are only GFP-positive. During the course of the timelapse, different events were observed that are all highlighted with colored circles. The red circle marks a haemogenic endothelial cell as it underwent basal endothelial to haematopoietic transition. In the mesenchyme, the cell divided once and the daughter cells remained more or less stationary. As time went on, haematopoietic cells in the mesenchyme lost their green fluorescence faster than their blue fluorescence. Cells that entered the vein to join the circulation were clearly less brightly green than cells that had only just undergone EHT. Based on the residual intensity of their green fluorescence, it was obvious that cells remained in the mesenchyme for different periods of time before they entered the vein. They also entered the vein by different routes. One cell was seen to migrate around the vein before entering it through its ventral wall (blue circle). Another cell entered the vein through its dorsal wall (green circle) while several other cells got trapped in an endothelial pocket formed by venous endothelial cells (yellow circle). The cells eventually left the pocket to join the circulation.

**Movie 5: Some flk1/kdrl:GFP;csl:Cer double-positive haemogenic endothelial cells leave the endothelium apically.** This movie supports findings reported in Figure 1. It shows data from timelapse confocal microscopy on *flk1/kdrl:gfp/csl:cer*-double transgenic embryos starting from 30 hpf. The arrow points at a haemogenic endothelial cell that leaves the endothelium apically to enter the dorsal aorta. The embryo was immobilized in 1% low melting point agarose. The images show single 1.5 μm thick optical sagittal sections of an embryo with anterior to the left and dorsal up. During the timelapse, images were taken every 3 minutes.

**Movie 6: qmc551:GFP-positive haematopoietic cells seed perivascular niches in the caudal haematopoietic tissue at 2 dpf and patrol the head tissues at 6 dpf.** This movie is related to Figure 3. **(A)** Timelapse confocal microscopy on *qmc551;flk1:tdTom* double transgenic embryos starting from 48 hpf showing 2.0 μm thick optical slices through the caudal haematopoietic tissue. Here, GFP+ haematopoietic cells undergo dynamic interactions with tdTom+ ECs. One of the cells is highlighted with a yellow circle. Please note that ECs in the ventral wall of the caudal artery co-express the two transgenes. Anterior is to the left, dorsal is up. The embryo shown was immobilized in 1% low melting point agarose. **(B)** A 6 dpf *qmc551* transgenic embryo was anesthetized and placed in a tiny depression in agarose under a fluorescent Nikon SMZ1500 dissection microscope using a Nikon DS-5Mc/DS-U1 camera setup. From minute 5 on, pictures of the head region of the embryo (facing right) were taken manually every 3 min. Images were imported into Photoshop. In Photoshop, the pictures were moved and rotated to correct for the drifting movement of the embryo under the microscope. Furthermore, annotations were added. All pictures were then imported into iMovie to generate the final video. Individual frames are also shown in Figure 3O.

**References**

**Ellett, F., Pase, L., Hayman, J. W., Andrianopoulos, A. and Lieschke, G. J.** (2011). mpeg1 promoter transgenes direct macrophage-lineage expression in zebrafish. *Blood* **117**, e49–56.

**Flicek, P., Amode, M. R., Barrell, D., Beal, K., Billis, K., Brent, S., Carvalho-Silva, D., Clapham, P., Coates, G., Fitzgerald, S., et al.** (2014). Ensembl 2014. *Nucleic Acids Res* **42**, D749–55.

**Gering, M. and Patient, R.** (2005). Hedgehog signaling is required for adult blood stem cell formation in zebrafish embryos. *Dev Cell* **8**, 389–400.

**Gray, C., Packham, I. M., Wurmser, F., Eastley, N. C., Hellewell, P. G., Ingham, P. W., Crossman, D. C. and Chico, T. J.** (2007). Ischemia Is Not Required for Arteriogenesis in Zebrafish Embryos. *Arteriosclerosis, Thrombosis, and Vascular Biology* **27**, 2135–2141.

**Itoh, M., Kim, C.-H., Palardy, G., Oda, T., Jiang, Y.-J., Maust, D., Yeo, S.-Y., Lorick, K., Wright, G. J., Ariza-McNaughton, L., et al.** (2003). Mind bomb is a ubiquitin ligase that is essential for efficient activation of Notch signaling by Delta. *Dev Cell* **4**, 67–82.

**Jin, S.-W., Beis, D., Mitchell, T., Chen, J.-N. and Stainier, D. Y. R.** (2005). Cellular and molecular analyses of vascular tube and lumen formation in zebrafish. *Development* **132**, 5199–5209.

**Sieger, D., Tautz, D. and Gajewski, M.** (2003). The role of Suppressor of Hairless in Notch mediated signalling during zebrafish somitogenesis. *Mech Dev* **120**, 1083–1094.

**Traver, D., Paw, B. H., Poss, K. D., Penberthy, W. T., Lin, S. and Zon, L. I.** (2003). Transplantation and in vivo imaging of multilineage engraftment in zebrafish bloodless mutants. *Nat Immunol* **4**, 1238–1246.

**Weinstein, B. M., Schier, A. F., Abdelilah, S., Malicki, J., Solnica-Krezel, L., Stemple, D. L., Stainier, D. Y., Zwartkruis, F., Driever, W. and Fishman, M. C.** (1996). Hematopoietic mutations in the zebrafish. *Development* **123**, 303–309.

**List of Abbreviations**

(excluding widely used abbreviations and names of genes and gene products)

ALM anterior lateral mesoderm

bEMT basal epithelial to mesenchymal transition

CHT caudal haematopoietic tissue

DA dorsal aorta

EC endothelial cell

EHT endothelial to haematopoietic transition

EMP epithelial to mesenchymal transition

HC haematopoietic cell

HEC haemogenic endothelial cell

HPC haematopoietic progenitor cell

hpf hours post fertilization

HSC haematopoietic stem cell

HSPC haematopoietic stem and progenitor cell

ICM intermediate cell mass

KM kidney marrow

PBI posterior blood island

PCV posterior cardinal vein

PLM posterior lateral mesoderm

prRBCs primitive red blood cells

RBCs red blood cells

vCA ventral wall of the caudal artery

vDA ventral wall of the dorsal aorta

WISH whole-mount in situ hybridisation
